# Passive Polarized Vision for Autonomous Vehicles: A Review

**DOI:** 10.3390/s24113312

**Published:** 2024-05-22

**Authors:** Julien R. Serres, Pierre-Jean Lapray, Stéphane Viollet, Thomas Kronland-Martinet, Antoine Moutenet, Olivier Morel, Laurent Bigué

**Affiliations:** 1 The Institute of Movement Sciences, Aix Marseille University, CNRS, ISM, CEDEX 09, 13284 Marseille, France; stephane.viollet@univ-amu.fr (S.V.); thomas.kronland-martinet@univ-amu.fr (T.K.-M.); antoine.moutenet@safrangroup.com (A.M.); 2 Institut Universitaire de France (IUF), 1 Rue Descartes, CEDEX 05, 75231 Paris, France; 3 The Institute for Research in Computer Science, Mathematics, Automation and Signal, Université de Haute-Alsace, IRIMAS UR 7499, 68100 Mulhouse, France; pierre-jean.lapray@uha.fr; 4 Materials Microelectronics Nanosciences Institute of Provence, Aix Marseille University, Université de Toulon, CNRS, IM2NP, 13013 Marseille, France; 5 Safran Electronics & Defense, 100 Av. de Paris, 91344 Massy, France; 6 ImViA, Laboratory, University of Bourgogne, 71200 Le Creusot, France; olivier.morel@u-bourgogne.fr

**Keywords:** bio-inspired vision, multi-modal vision, unconventional vision, scene understanding, linearly polarized light, passive polarization sensing, celestial compass, polarized geolocation

## Abstract

This review article aims to address common research questions in passive polarized vision for robotics. What kind of polarization sensing can we embed into robots? Can we find our geolocation and true north heading by detecting light scattering from the sky as animals do? How should polarization images be related to the physical properties of reflecting surfaces in the context of scene understanding? This review article is divided into three main sections to address these questions, as well as to assist roboticists in identifying future directions in passive polarized vision for robotics. After an introduction, three key interconnected areas will be covered in the following sections: embedded polarization imaging; polarized vision for robotics navigation; and polarized vision for scene understanding. We will then discuss how polarized vision, a type of vision commonly used in the animal kingdom, should be implemented in robotics; this type of vision has not yet been exploited in robotics service. Passive polarized vision could be a supplemental perceptive modality of localization techniques to complement and reinforce more conventional ones.

## 1. Introduction

Navigating in Global Navigation Satellite System-denied or unmapped environments will, over the coming decade, become one of the 10 biggest challenges in robotics [1]. Currently, autonomous robots rely on Global Navigation Satellite System (GNSS), Inertial Navigation Systems (INS) and ground-based antennas to triangulate or correct GNSS signals (5G networks or Real-Time Kinematic (RTK) networks), astronomical navigation, gyrocompass navigation, and vision-based or lidar-based SLAM (SLAM stands for Simultaneous Localization And Mapping). Surprisingly, passive polarized vision has not yet become standard in robotics to improve the SLAM technology brick, geolocation, or true north heading detection, for instance. In contrast, animals are able to navigate or migrate over extremely long distances without using localization techniques developed by humans [2]. Migratory birds should be mentioned: they are known for their astonishing navigation capabilities. Studies have shown that some of them, such as Savannah sparrows [3] or *Catharus* thrushes [4], can navigate by means of a Polarization-based Compass (PbC), which they use to calibrate their magnetic compass. However, the precise mechanism involved in this calibration remains unclear [5]. On the other side, how insects use sky polarization to navigate is better understood. For instance, desert ants use a powerful navigational tool termed optical path integration to locate their nest. When returning, desert ants follow the shortest possible route—a straight line—even in featureless or unfamiliar terrains. By integrating a directional compass and distance information from their vision, desert ants calculate a vector from their visual inputs, and this leads them home [6]. Since pioneering behavioral experiments on desert ants by Piéron (1904) [7], who manipulated ant position in order to observe their behavior, and Santschi (1911) [8], who manipulated the light perceived by ants by using a mirror, it has taken about a century to understand how desert ants exploit sunlight for navigation purposes [6,9]. The ant-inspired path integrator has been recently implemented on board fully autonomous robots: firstly, a wheeled robot, called Sahabot in 2000 [10], then a legged robot, called AntBot in 2019 [11,12] (see Section 3.3 for further information).

**Figure 1:**
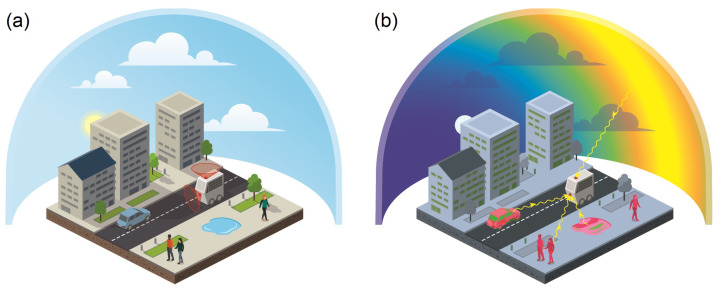
Illustration of the available polarized light in the environment. Picture credits: Camille Dégargin (2023). (**a**) Visual environment as seen by the robot with unpolarized light, i.e., light intensity. (**b**) Visual environment as seen by the robot with polarized light, which can be either due to the light scattering from the sky or the light reflection from surrounding environment.

Understanding how roboticists can exploit polarized sunlight or light reflection is at an early stage (Figure 1), but it is extremely relevant because this could pave the way for the development of a GNSS-free geolocation for autonomous outdoor navigation as it works in the animal kingdom [13]. It should be noted that long before the modern polarization sensing systems described below, between the 10th and 13th century, Viking navigators had been using skylight polarization for navigation to reach Greenland and then North America without a magnetic compass, instead using a sunstone working as an optical compass (see [14,15,16,17] and Section 3.1 for further details).

This review article was written to address common research questions in the field of autonomous robotics:

- What kind of polarization sensing can we embed into robots? (see Section 2)
- Can we geolocate ourselves and find the true north heading by detecting light scattering from the sky? (see Section 3)
- How do polarization images relate to the physical properties of reflecting surfaces in the context of scene understanding? (see Section 4)

Autonomous robots working in urban environments, e.g., for last-mile delivery services, will have to locate and position themselves with a spatial accuracy of better than 5 cm and 0.2 degrees by 2030. Mobile robots navigating through public environments (in urban areas or on a campus for instance, see Figure 1a) must meet the most stringent safety requirements. They must comply with the Machine Directive (ISO 3691-4 [18]), as well as autonomous vehicle standards such as Directives ISO 26262 [19] (Fusa) and ISO 21448 [20] (Sotif). Using and fusing the polarized sensors’ outputs with an Inertial Navigation System (INS) could be a supplemental perceptive modality of localization techniques that would help reach the level of performance required by ISO standards in order to complement and reinforce the conventional localization techniques (3D LiDAR-based SLAM, GNSS, and visual–inertial odometry).

Robots will therefore use all the available visual information including that coming either from the light scattering of the sky or the light reflection from surrounding environment. Even if the sun is hidden or the sky is covered, light scattering remains available, and this includes relevant and robust information for robots’ navigation (Figure 1b). Moreover, using polarized light reflection will be useful to improve visual contrast in order to better understand the visual scene through superior object detection.

To help researchers find relevant directions over the next decade in the field of passive polarized vision in autonomous vehicles, we will divide this review into three main sections. Following this logic, the three main sections are as follows: Section 2, “Embedded polarization sensing”, focusing on polarimetric sensors which can be embedded on board robots; Section 3, “Polarized vision for navigation”, which will emphasize how polarized light scattering can be used for navigation purposes; and lastly Section 4, “Polarized vision for scene understanding”, which will suggest how polarized light reflection can be used to better understand a visual scene. Figure 1 illustrates the links between these three sections as they relate to the above main research questions. To address each of these questions, it is required to divide them into more specific research questions on how we can transfer knowledge derived from the physical properties of polarized light to sensors and then use it for navigation purposes.

Previous review articles have focused on the progress of bio-inspired polarized sensors and comprised an exhaustive overview on polarized sensors manufactured by nanotechnology [21], polarization based orientation estimation algorithms, and the combination of polarized sensors with INS, GNSS, SLAM, and other localization systems [22,23] or polarization-based geolocation [23,24] instead of being focused on passive polarized vision in autonomous vehicles in which the dynamic accuracy is more relevant than the static accuracy. Despite the growing interest in bio-inspired polarized sensors for navigation purposes, few of them have been implemented on board mobile robots.

In Section 2, we will introduce the various technological solutions to embed polarization imaging into robots. Current state-of-the-art polarization acquisition techniques will be introduced. Only relevant technologies, comprising passive and linear Stokes polarimetry, in the framework of mobile robotics will be presented in this section. Section 2 will be divided into following five subsections:

- Section 2.1. Stokes formalism;
- Section 2.2. State-of-the-art of polarization analysis techniques;
- Section 2.3. Calibration and preprocessing;
- Section 2.4. Extension to multispectral polarimetric sensing;
- Section 2.5. Summary and future directions in embedded polarization sensing.

In Section 3, we will introduce the various implementations of polarization-based navigation systems on board autonomous robots. Starting with a brief historical overview of polarization navigation, we will then describe the skylight polarization pattern from the simplest model to the most advanced ones. Next, we will present an exhaustive overview of polarized sensors implemented on board vehicles or autonomous robots for heading or attitude estimation. Lastly, recent developments in polarization-based geolocation will be presented. Section 3 will be divided into the following five subsections:

- Section 3.1. Historical overview of polarization navigation;
- Section 3.2. The skylight’s polarization pattern;
- Section 3.3. Polarization-based sensors dedicated to navigation;
- Section 3.4. Methods for combining polarization-based geolocation to an integrated navigation system;
- Section 3.5. Summary and future directions in polarized vision for navigation.

In Section 4, we will introduce the use of polarized vision for scene understanding. Polarization has been widely used in the classification of materials [25] or reconstruction of object shape [26]: the section will only focus on applications that can be directly extended to autonomous vehicles. After recalling and explaining all the mathematical formula linking the polarization parameters to the normal orientation, object detection will be described. Then, shape from polarization that exploits most of the physical information and the latest techniques using polarization imaging to improve depth estimation and facilitate pose estimation in robots will be presented . Section 4 will be divided into the following five subsections:

- Section 4.1. Polarization and reflection;
- Section 4.2. Detection and classification;
- Section 4.3. Shape from polarization;
- Section 4.4. 3D-depth with polarization cues;
- Section 4.5. Summary and future directions in polarized vision for scene understanding.

In Section 5, we will deal with lessons learned from this review and provide new lines of research and future directions in the sensing of polarized light in robotics for the next 10 years.

## 2. Embedded Polarization Imaging

We present here the state of the art on polarimetric techniques that allow for the capture of the polarization characteristics of an unknown beam of light. We will first consider Stokes formalism. We focus on technologies that seem appropriate for the framework of on-board acquisition systems in mobile robotics: passive and linear Stokes imaging polarimeters. Non-imaging sensors used for navigation are detailed in Section 3.3. Further information about point-source sensors can be found in [21].

### 2.1. Stokes Formalism

**Figure 2:**
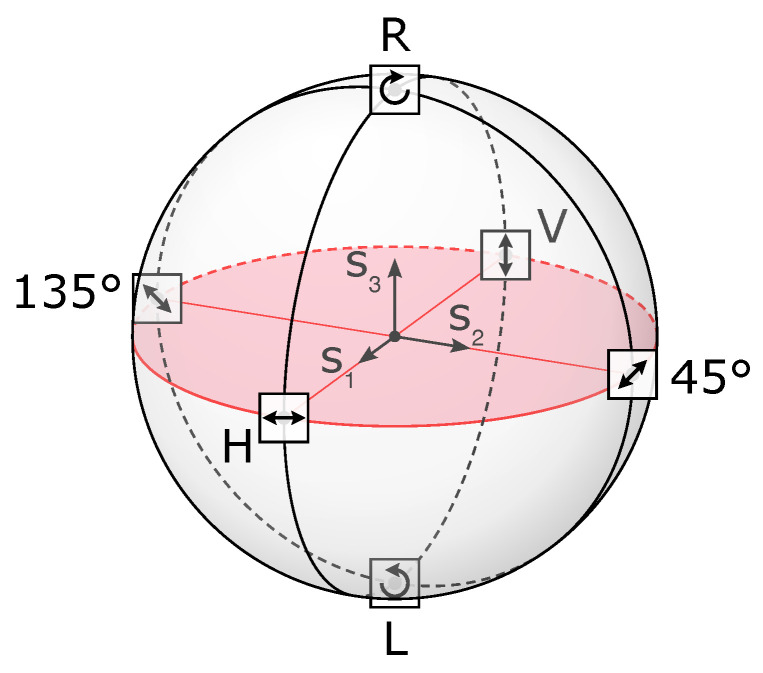
Poincaré sphere. In the equatorial plane (in pink), we can find purely linear polarizations that are considered in our review. Adapted from original material under CC-BY license [30].

The linear polarization state of light depends on the material properties of the objects in the scene, but also on the geometry of the incident light beam (angle of incidence and angle of reflection), and on the state of polarization of the incident light. The whole polarization information about the scene is contained in the four-component Stokes vector $S={\left[s_{0}\;s_{1}\;s_{2}\;s_{3}\right]}^{T}$, sometimes referred to as $S={\left[I\;Q\;U\;V\right]}^{T}$ [27,28]. The first component is related to the total energy in the scene (polarized or not), the second and third components are related to the linear polarization, and the fourth component is related to circular polarization. A convenient representation of the Stokes vector is the Poincaré sphere [29], such as described Figure 2.

The effect of any optical change on an input Stokes vector $S_{\mathrm{in}}$ into an output vector $S_{\mathrm{out}}$ can be described by a 4×4 Mueller matrix M such as [31,32]:

(1) $$S_{\mathrm{out}}=M.S_{\mathrm{in}}$$

Mueller matrix estimation can be used to study and classify materials [33,34,35,36,37] or for biomedical applications [38,39]; however, in the following, we will restrict ourselves to Stokes estimation.

In outdoor robotic conditions, i.e., in environments with passive illumination, beams with significant elliptical polarization are rarely encountered since first diffusion only produces linear polarization [40,41]. For practical purposes, we will therefore limit ourselves to linear polarization for the description of Stokes formalism and thus consider that $s_{3}=0$, which should be confirmed for each application.

Estimating the linear Stokes vector can be performed through the measurement of foujr elementary intensities measured through a linear polarizer oriented at 0°, 45°, 90° and −45° and therefore named $I_{0}$, $I_{45}$, $I_{90}$, and $I_{-45}$:

(2) $$\begin{array}{c} S=\left[\begin{array}{c} s_{0}\\ s_{1}\\ s_{2}\\ 0 \end{array}\right]=\left[\begin{array}{c} I_{0}+I_{90}\\ I_{0}-I_{90}\\ I_{45}-I_{-45}\\ 0 \end{array}\right] \end{array}$$

This is probably the most popular method for capturing linear Stokes parameters. However, reduced schemes using only three measures also exist [42]. It should be noted that the choice of configuration affects the system condition number, which impacts on performance metrics such as the signal-to-noise ratio (SNR) [43], specifically and thoroughly in ref. [44] for a four-polarizer filter array sensor. Moreover, it has been demonstrated that the polarization angles used for the polarization state analyzer (PSA) that minimize noise influence form a regular polyhedron in the Poincaré space (which is a unit disk for linear polarization or a unit sphere in the general case) [42].

It should be noted that S is not an algebraic vector (no additive inverse, for instance) and that any vector in $R^{4}$ is not a Stokes vector. Stokes vector components must fulfill:


{(3)s0≥0(4)s02≥s12+s22+s32

A generalized measurement framework can be derived for any polarimeter using *N* polarization channels. The principle is to perform intensity measurements in *N* different configurations of the PSA after proper calibration, meaning proper determination of the *N* analyzer vectors. Since in our case $s_{3}=0$, we can write the following:

(5) $$\begin{array}{c} I=\left[\begin{array}{c} I_{1}\\ I_{2}\\ \vdots \\ I_{N} \end{array}\right]=W.S=\left[\begin{array}{cccc} A_{1,0} & A_{1,1} & A_{1,2} & 0\\ A_{2,0} & A_{2,1} & A_{2,2} & 0\\ \vdots & \vdots & \vdots & \vdots \\ A_{N,0} & A_{N,1} & A_{N,2} & 0 \end{array}\right]\left[\begin{array}{c} s_{0}\\ s_{1}\\ s_{2}\\ 0 \end{array}\right]\; \end{array}$$

where W is the polarimetric measurement matrix formed by the analyzer vectors. Provided the *N* configurations are properly chosen, the Stokes vector is estimated by using $\hat{S}=W^{+}I$, where $W^{+}$ is often called the Data Reduction Matrix (DRM) or the analysis matrix, computed using the pseudo-inverse method [45,46].

One can derive polarization metrics from the linear Stokes vector, for instance the Degree of Linear Polarization DoLP:

(6) $$DoLP=\frac{\sqrt{s_{1}^{2}+s_{2}^{2}}}{s_{0}}$$

and the Angle of Linear Polarization AoLP:

(7) $$AoLP=\frac{1}{2}\mathrm{atan}2\left(s_{2},s_{1}\right)$$

Both DoLP and AoLP are useful for skylight navigation, as detailed in Section 3.2. In many cases, authors often mention using the Degree of Polarization DoP:

(8) $$DoP=\frac{\sqrt{s_{1}^{2}+s_{2}^{2}+s_{3}^{2}}}{s_{0}}$$

In our specific case, with no circular polarization, evaluating DoP comes down to evaluating DoLP.

### 2.2. State-of-the-Art Polarization State Analyzers (PSA)

Two main categories of Polarization State Analyzers (PSAs) exist, both of them providing Stokes information: scanning and snapshot systems. All Division-of-Time (DoT) polarimeters belong to the class of scanning instruments, i.e., several sequential acquisitions are needed to obtain the polarimetric information, but some of these are fast enough to be compatible for robotic applications. Thus, we first present the DoT techniques, and then present the snapshot techniques, namely Replication of Aperture (RoAp), Division of Amplitude (DoAmp), Division of Aperture (DoAp) and Division of Focal Plane (DoFP).

Table 1, inspired by that of Tyo et al. [47], lists the various polarimetric imaging techniques detailed below. It is worth noting that we only include the technologies that permit the capture of two to four Stokes parameters in an efficient way.

#### 2.2.1. PSA Using Division of Time (DoT)

##### PSA with Rotating Polarization Elements

Conceptually, this is the simplest kind of PSA. A single rotating element (a polarizer or a waveplate in front of a fixed polarizer) is located in the optical path between the object and the sensor. *N* azimuth angles are considered for the rotating element, as in Equation (5). The maximum rank for the analysis matrix W is 3, even if we multiply the measurements in various angular configurations. Only a partial Stokes vector is therefore analyzed. To analyze all the Stokes information, it is necessary to use as a polarization element an extra waveplate whose retardance is not an integer multiple of λ/2. Typically, a quarter-wave plate is used. Tyo [43] showed that a waveplate with a retardance of 0.3661λ optimizes the system SNR (provided that optimum angles are chosen). This type of assembly, whether the waveplates are motorized or not, remains slow, but does potentially present the best optical quality. However, it may be necessary to register the different images before calculating the Stokes parameters (see Section 2.3.2). Such techniques have been successfully used for skylight polarization estimation [48,49,50].

##### PSA Using Liquid Crystal Cells

It is advantageous to replace the mechanically rotating polarization element with an electrically controlled liquid crystal cell. You can consult [71] for an overview of the physics and use of these liquid crystals. Due to Wolff [51], this idea gave rise to many works in the 2000s. In this preliminary implementation, polarization is rotated thanks to two twisted nematic liquid crystal cells controlled in a binary manner; therefore, a maximum of four directions of analysis is obtained. It is reasonable to estimate that the rate of 25 frames per second (fps) can be reached. Gandorfer accelerated this setup thanks to ferroelectric liquid crystal cells (smectic C) [53], thus achieving 250 fps. Some experimental works improved this principle using a single tunable ferroelectric liquid crystal cell which allows a continuous adjustment of the polarization rotation [56,72] and obtaining three Stokes parameters. Optimized use of modulator control and chromatism also achieves the fourth Stokes parameter, but at the cost of higher noise [73].

Commercial liquid crystal polarimetric cameras were marketed as early as the 2000s by BossaNova [57,58]. Today, cameras based solely on liquid crystal modulators are no longer an optimum solution and have mainly been replaced by Polarimetric Filter Array (PFA) cameras (see Section 2.2.5) when only the linear polarization is of interest. However, liquid crystal modulators remain interesting for analyzing circular polarization [74] (impossible for commercial PFA cameras) and also for generating polarization states in Mueller imaging as Polarization State Generators.

Liquid crystal techniques for skylight observation were reported in Refs. [55,59].

#### 2.2.2. PSA Using Replication-of-Aperture (RoAp)

These systems are conceptually very straightforward: you place as many cameras and optics as you need next to each other. Systems dedicated to three Stokes parameters, measuring along three or four polarization directions, were reported [60,61,62]. These systems are rather expensive and require both a calibration of the different systems and a registration of the different polarization images before estimating Stokes parameters.

#### 2.2.3. PSA Using Division of Amplitude (DoAmp)

##### Use of Beam Splitters

This type of assembly is conceptually quite simple, since it consists of dividing the beam into as many sub-beams as there are measurements to be performed. In practice, if one wants to access all the Stokes information, this leads to relatively heavy setups, since four analysis arms are required. The optical elements must be of high quality and the images must be mechanically or digitally registered. An apparently high-performance compact version has been proposed [63]; it allows access to all the linear polarization information. A simplified version consists of placing oneself in a monostatic configuration and analyzing only two crossed polarization components by dividing the beam using a Wollaston prism. The latter makes it possible to shift the two polarization components and thus juxtapose them on the detector [64]. It should be noted that a version providing full Stokes information has already been implemented in the infrared [75].

A noteworthy approach combining DoT and DoAmp has been suggested [65]. It forms Stokes components calculated from images acquired simultaneously, therefore with a reduced shift between the different images. In this case, only the first three Stokes components are considered, and two measurement arms are used preceded by a ferroelectric liquid crystal modulator acting as a rotator. Thus $\left(I_{0},I_{90}\right)$ are simultaneously acquired, and afterwards $\left(I_{45},I_{-45}\right)$. An approach combining DoA and DoFP is also possible [76].

##### Use of PGA

The measurement of the different polarization components can also be performed via Polarization Gratings Arrays (PGAs) [77,78]. They are composed of anisotropic diffraction optical elements to spatially separate polarization information. PGAs have the property of producing chromatic dispersion proportional to the polarization state of the light, generating a pattern that can be focused and captured on a focal plane. This technique has the advantage of capturing polarization information using a spectral band with a spectral resolution down to 1 nm [79] and allows spectropolarimetric imaging with a simple and compact design.

#### 2.2.4. PSA Using Division of Aperture (DoAp)

This technique is rather similar to the division-of-amplitude method, but the system is more compact since it uses only one camera [66] at the expense of a loss of definition in the polarization images. The optical system is also more complex. It was implemented in the middle-wave infrared but could be considered in the visible range [66].

#### 2.2.5. PSA Using Division of Focal Plane (DoFP)

**Figure 3:**
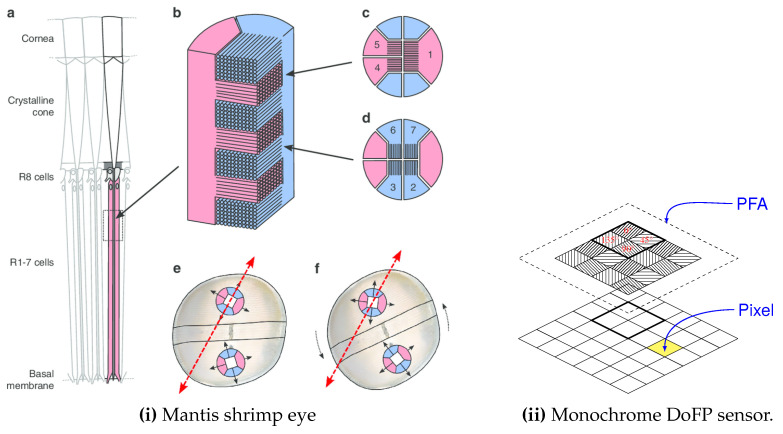
(**i**) Mantis shrimp eye is a good example of Division of Focal Plane as far as polarization is concerned (originally published in [81] and made available under CC-BY-SA license [82]). Subfigure (**i**).**a** highlights a rhabdom (in pink), which can be seen as a waveguide. The cornea acts as a sensor. A section of the rhabdom is shown in Subfigure (**i**).**b**, with retinular cells made of microvilli stacks (here coloured in red and blue), as described in Subfigures (**i**).**c** and (**i**).**d**. These microvilli act as polarizers. Since each rhabdom contains microvilli in crossed directions, each rhabdom allows selection of two crossed polarizations. Since rhabdoms are shifted by 45° between the ventral and dorsal hemispheres as depicted in Subfigures (**i**).**e** and (**i**).**f**, the eye can actually sense 4 equally spaced directions of polarization. In Subfigure (**i**).**e**, polarization direction (red arrow) is aligned with a set of microvilli in the dorsal hemisphere, so the polarization direction is easily detected. In Subfigure (**i**).**f**, the eye has rotated by 22.5°; polarization direction (red arrow) is aligned with none of the sets of microvilli in the dorsal or ventral hemispheres, so the eye cannot detect the polarization direction. Subfigure (**ii**) describes a modern polarization-sensitive camera sensor, such as Sony Polarsens IMX264MZR, which mimics the mantis shrimp eye, with micropolarizers with different orientations placed side by side in front of the photosensitive sensor.

**Figure 4:**
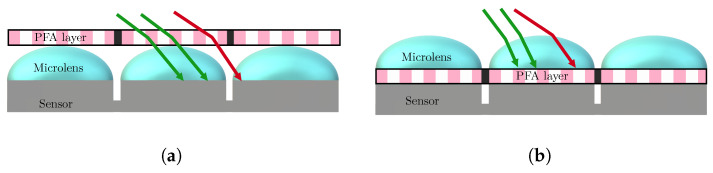
Two assembly schemes for PFA integration: on-glass (**a**) and on-chip (**b**) schemes. In both schemes, most rays (depicted as green arrows) hit the right pixel. For the on-glass scheme, some oblique rays (red arrows) may hit the wrong pixel, which is not possible with the on-chip scheme. Therefore the on-chip scheme used in PolarSens Sony Sensors, with the PFA between the microlenses and the sensor, greatly reduces polarimetric crosstalk. Reproduced with permission from Yilbert Gimenez [83].

The idea reported Figure 3 takes up that already proposed by Bayer for RGB cameras [80]: the pixels do not all capture the same state of polarization. An array of microfilters (aluminum nanowires), often referred to as Polarizer Filter Array (PFA) composed of a pattern of four pixelated polarizers which are repeated many times on the grid, is placed in front of the sensor. These four polarizers allow capturing vertical, horizontal, 45° and −45° linear polarizations. This idea, proposed by [67] and implemented in particular by Gruev et al. [68], has been commercially developed by 4D Technology [69] and especially Sony Semiconductors which provide sensors to camera integrators [70]. Tremendous progress has been made with this technology over the past ten years. Whereas 4D technology puts the PFA on top of the microlens array, Sony puts the PFA between the microlens array and the sensor itself, which greatly reduces polarization crosstalk, as described Figure 4.

The cameras based upon Sony PolarSens sensors proved to be very successful in the scientific community and have been the subject of much literature concerning their characterization and calibration [84,85,86,87,88,89] and demosaicing preprocessing [90,91,92,93]. Among other things, they have enabled the development of applications for driving assistance [94,95] and autonomous navigation [96].

Commercially available cameras have resolutions of 5 to 12 Mpixels and provide 12-bit information with moderate noise for less than $1500. Depending on their communication interface, they can be operated up to 90 fps. If we stick to the acquisition of linear polarization, they have supplanted the DoT and DoA technologies. Since the operating rate only depends on the sensor technology, PFA cameras able to operate up to 7000 fps are reported [97,98]. To acquire the complete Stokes vector, one can combine two PFAs (one of which is equipped with a retarder waveplate) [76] in a hybrid DoA–DoFP architecture or place a liquid crystal modulator in front of the camera (DoT–DoFP architecture) [99]. A laboratory device acquiring the full Stokes information using a single PFA has been proposed [100].

In DoFP PFA systems, the most common polarization arrangement is a 2×2 repeating pattern of analyzers and has been introduced by Chun et al. [67]. Other spatial arrangement patterns for micropolarizers have been found to be less sensitive to visual artifacts in the reconstructed images [101,102,103], but none have been implemented in a camera to our knowledge.

### 2.3. Calibration and Preprocessing Operations

The aforementioned hardware systems, whatever their characteristics, provide raw data that must be preprocessed in order to be used for navigation operations. Such raw data, without any corrections or preprocessing, usually result in polarimetric data full of artifacts.

#### 2.3.1. PSA Calibration

To precisely estimate the Stokes vector from intensity measurements, W must be estimated very accurately. This compensates for the imperfect polarization optics, i.e., the transmission, diattenuation, and polarization angle characteristics. A first solution consists of the component-wise calibration using a reference metrology polarimeter [104]. Another popular solution proves to be a block calibration of the whole system from the camera responses. It consists of generating a set of *M* well-known reference polarization states using an ‘ideal’ polarizer—the Stokes vectors of which are gathered in a matrix named $S_{m}$—and taking a set of *N* measurements with the PSA for each of the reference states gathered in a matrix named $I_{m}$. Therefore, we can write the following:

(9) $$\begin{array}{c} I_{m}=\left[\begin{array}{cccc} I_{1,1} & I_{1,2} & \cdots & I_{1,M}\\ I_{2,1} & I_{2,2} & \cdots & I_{2,M}\\ \vdots & \vdots & \ddots & \vdots \\ I_{N,1} & I_{N,2} & \cdots & I_{N,M} \end{array}\right]=W.S_{m}=W\left[\begin{array}{cccc} s_{0,1} & s_{0,1} & \cdots & s_{0,M}\\ s_{1,1} & s_{1,1} & \cdots & s_{1,M}\\ s_{2,1} & s_{2,1} & \cdots & s_{2,M}\\ 0 & 0 & \cdots & 0 \end{array}\right]\; \end{array}$$

An estimate of W can then be computed using the pseudo-inverse method [45,46] by $\hat{W}=I_{m}S_{m}^{+}$ and thus the Stokes vector is estimated by using $\hat{S}={\hat{W}}^{+}I$, where ${\hat{W}}^{+}$ is often called the Data Reduction Matrix (DRM). Alternative estimators have also been considered, like Singular Value Decomposition (SVD) [105] or the Eigenvalue Calibration Method (ECM) [106]. Some works assume that the polarization measurement is mainly affected by signal-dependent Poisson shot and Gaussian noise. These considerations have been used to select the optimal reference polarization states to take into account both Poisson and Gaussian noises [85,107,108].

Depending on the type of PSA, calibration may be simplified; for instance, a CCD camera equipped with a rotating polarizer may not require a pixel-to-pixel characterization. A polarimeter including a liquid crystal (LC) cell will require a careful characterization of the LC cell (which behavior may depend on wavelength and temperature). A PFA CMOS camera may require a full characterization since both the CMOS sensor and the PFA exhibit pixel-to pixel variations [109].

Calibration methods have been specifically designed for DoFP PFA polarimeters, like the super-pixel method [84,87,110], which jointly calibrates a group of 2×2 pixels instead of calibrating independently each pixel. A recent study evaluated the efficiency of this method for extreme camera lens configurations (focal lengths and apertures) [89]. Other evolved calibration methods have been studied which do not need precise and cumbersome instruments [111,112] or spatially uniform illumination [85]. In some cases, for instance if the camera is used in an 8-bit mode, especially when it is based on a Sony PolarSens sensor, a single overall calibration may be sufficient. In this case, only the average figures of the transmission ratio and the orientation angle are considered over the whole image [113].

#### 2.3.2. Spatial Reconstruction of DoFP Images

In the case of DoFP polarimeters, there is a spatial sampling of the analyzers, i.e., the focal plane array is spatially modulated. Thus, each pixel senses only a specific polarization state of a specific point in the scene. A very easy solution consists of subsampling the original raw image into four linear polarization direction images, but this operation produces Instantaneous Field of View (IFoV) errors resulting in strong artifacts in DoLP and AoLP images. A registration seems mandatory [114,115]. An alternative consists of reconstructing directional images produced by PFA cameras to their full resolution, in order to avoid possible interpretation errors by computer vision algorithms. This aims to estimate the values of each of the missing polarization channels at a pixel location. The operation is called demosaicing, and has been extensively studied in the literature, especially for RGB Bayer microfilter patterns.

As in the spectral case, demosaicing algorithms can benefit from several assumptions, like spatial correlation or polarization channel correlations. Nevertheless, several algorithms are dedicated to DoFP, including the physical constraint $I_{0}+I_{90}=I_{45}+I_{135}$ [116]. These are based either on filtering [117,118], adaptive filtering [119,120], linear systems theory [114], motion [121,122], or learning approaches [123,124,125].

#### 2.3.3. Spatial Registration of Elementary Polarization Images

Many polarimeters exhibit a spatial shift between the various polarization channels. It can be due to an imperfect alignment (Division of Amplitude polarimeters), to imperfect components, for instance wedge effect (Division of Time polarimeters), or to a rigid relative motion between the camera and the scene (Division of Time polarimeters). This shift, even if it is a subpixel shift, is likely to produce strong artifacts in DoLP images for instance [126]. Efficient solutions can be applied to circumvent this phenomenon [127,128].

Images produced by PFA DoFP cameras also exhibit a shift when subsampled without demosaicing (see Section 2.3.2) and should also be registered [129].

Images produced by scanning polarimeters exhibit polarization artifacts at the edge of moving objects. This can be solved by optical flow techniques [130], but this solution remains rather computer intensive [131]. For this very reason, DoFP PFA polarimeters are now extremely popular, much more than DoT polarimeters.

#### 2.3.4. Denoising Polarization Images

As with any imaging system, an imaging polarimetric system is likely to be affected by noise. An abundant amount of works in the literature deal with this; a starting point could be Refs. [43,132,133,134]. This noise can be due to physics (Poisson noise) or due to imperfect or miscalibrated instruments (Gaussian noise, shift between polarimetric bands, etc.). Modern PSAs, provided they are carefully used and carefully calibrated, can produce intensity images with a reduced noise level, but the polarimetric pipeline leading to DoLP and AoP estimation may amplify this noise [135]. Recently, a metric named Accuracy of Polarization Measurement Redundancy (APMR) was proposed to quantify this polarimetric noise [136] regarding intensity images, but it is not necessarily correlated to noise affecting Stokes parameters.

To reduce noise, temporal averaging of intensity images is an obvious solution, although it is not always practicable. Filtering solutions originally dedicated to luminance or intensity imaging, such as BM3D [137], can be successfully adapted to polarization imaging, after Stokes estimation [138]. Another popular solution consists of using more than the minimum of three configurations of the PSA necessary to reconstruct the first three Stokes parameters, which is naturally possible for PFA DoFP commercial architectures which include four polarization directions by construction. It can be combined with other solutions consisting of calibrating the PSA (see Section 2.3.1 ) and if necessary compensating for IFoV errors (see Section 2.3.1).

### 2.4. Extension to Multispectral Polarimetric Sensing

The principles described earlier are compatible with broadband imaging, since linear polarizers usually exhibit a rather flat response over the visible range (and beyond). Nevertheless, when using waveplates or liquid crystal devices, a narrow spectral filter may be required since the retardance depends on the wavelength for such components, even for achromatic waveplates. Not taking this phenomenon into account results in errors in the estimation of DoFP [139].

**Figure 5:**
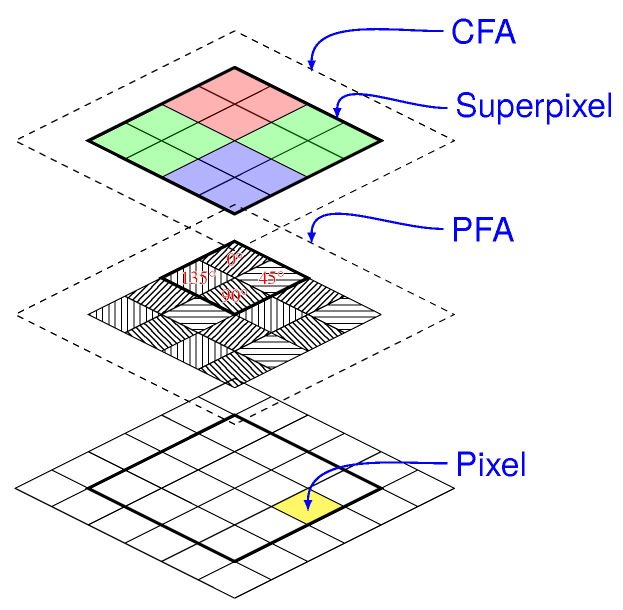
Color polarization filter array, such as those implemented in commercial sensors Sony IMX250MYR and IMX253MYR. An efficient demosaicing procedure is required.

Multispectral sensing can be considered, generally at the expense of further division of space or time [140]. In the first implementations, it consisted of a rotating wheel equipped with spectral filters [141,142]. As a snapshot alternative, in the past few years [143], color polarization filter arrays referred as CPFAs have been commercially released [70]: they mix two principles, the CFA (Color Filter Array) and the PFA, as described in Figure 5.

With such devices, we obtain 12-channel mosaiced images, the information is then rather sparse for each channel. Efficient demosaicing algorithms are required to prevent color and polarization reconstruction artifacts [125,144,145]. Alternate geometries combining CFA and PFA were proposed in order to maximize the signal to noise ratio or minimize the reconstruction artifacts [145,146,147].

### 2.5. Summary and Future Directions in Embedded Polarization Sensing

Efficiently capturing linear polarization in 2D in the field of autonomous navigation could benefit from several recent technological developments.

First, most snapshot polarimeters capture the filtered intensities in only one spectral band and in the visible part of the spectrum. This is no longer a real limit with color PFAs, at the expense of a loss in spatial resolution and an attenuation due to the use of spectral filters. This latter point has recently been overcome with a very promising solution consisting in using metasurfaces as routers [148]. The loss of spatial resolution could also be solved through the use of vertically stacked detectors, mimicking a mantis shrimp’s eye, as suggested and implemented by Garcia et al. [149,150] and Altaqui et al. [151]. This could enable the snapshot capture of spectropolarimetric information [152], which could be relevant to computer vision algorithms such as, for example, visibility restoration [153].

Second, robotic navigation using polarization sensing only considers linear polarization since skylight contains mainly linear polarization information. Using circular polarization may help, especially when navigation safety is concerned, as suggested by Geng et al. [154]. This will require that full Stokes snapshot PSAs are available. Several solutions could be considered, for instance using two PFA cameras [76], but promising solutions using only one camera also exist [155].

Finally, it seems that imaging sensors have already reached a mature level that has enlarged the audience of polarimetry. Open-source software toolkits such as Polanalyzer [156] and Pola4all [157] will be useful in the near future to help end users in robotics applications and beyond in the implementation of efficient solutions.

## 3. Polarized Vision for Robotics Navigation

### 3.1. Historical Overview of Polarization Navigation

Historically, Viking navigators are assumed to have been the first to exploit polarized light for navigation and exploration purposes. Viking navigators ruled the North Atlantic Ocean for about three centuries between about AD 900 and 1200. Their main sailing route was the 60°21′55″ latitude between Norway and Greenland. They used a sun compass to determine geographical north instead of a magnetic compass. It has been hypothesized that when the sun was invisible or below the horizon, Viking navigators determined the direction of polarization of skylight with sunstones—dichroic/birefringent crystals as polarizers—and then estimated the geographical north using the sunstone as a sun compass [14,15,16,17]. These research achievements suggest that the sky-polarimetric navigation is surprisingly effective on both days of the spring equinox and summer solstice, even under cloudy or foggy conditions. This sunstone-based compass explains why the Viking navigators could reach North America without a magnetic compass.

The United States, 750 years later, was the first to exploit this type of navigation for military purposes. In 1949, the US Army purchased four Pfund sky compasses to equip its Air Force [158]. During the Cold War, monitoring Alaska and crossing the North Pole to target the Union of Soviet Socialist Republics (USSR) was militarily vital, and magnetic compasses were not able to indicate a course. Lieutenant Commander Alton B. Moody reported that the Pfund sky compass had a heading accuracy of 1°. It was an optical instrument used manually by rotating a half-wave plate against a linear polarizing filter. In 1954, Scandinavian Airlines (SAS) launched the first scheduled passenger flight between Copenhagen (Denmark) and Los Angeles (USA), the world’s first polar shortcut using the Pfund sky compass principle. This new route reduced the travel time between California and Scandinavia from 36 to 22 h. SAS made further improvements and used the sky compass for many years on its polar flights. Since then, navigation based on polarimetric information has fallen by the wayside due to high-precision inertial navigation and GNSS navigation.

In the 2010s, a Sky Polarization Azimuth Sensing System (SkyPASS) emerged [159,160,161,162] as an American redevelopment of its own Pfund sky compass from the 1950s (see Section 3.3 **Military devices**). Since then, related studies relying on the polarized skylight for navigation purposes aim mainly to design and fabricate PbC for heading detection. As far we know, these studies were achieved by one American company developing the SkyPASS product (Polaris Sensor Technologies, Inc., USA, [160,161,162]), but they do not sell it outside of the USA (cost in excess of USD 10k), one research group in the USA [163], two French universities [11,12,164,165], one research group in Germany [166], one research group in Scotland [167], and seven Chinese universities [168,169,170,171,172,173,174,175,176,177,178,179,180,181,182,183].

### 3.2. The Skylight Polarization Pattern

The most common way to describe atmospheric scattering near the visible spectrum is the Rayleigh model (1871, [184,185]), which describes all the electromagnetic field properties by considering single elastic scattering by particles much smaller than the wavelength [186,187,188]. In the Rayleigh scattering model, there are two points featuring null DoLP: the sun direction and the anti-sun direction. However, atmospheric turbidity and multiple scattering causes differences between the Rayleigh scattering model and skylight polarization, which limits its trustworthiness and its use in robotics to determine an accurate celestial heading [189].

**Figure 6:**
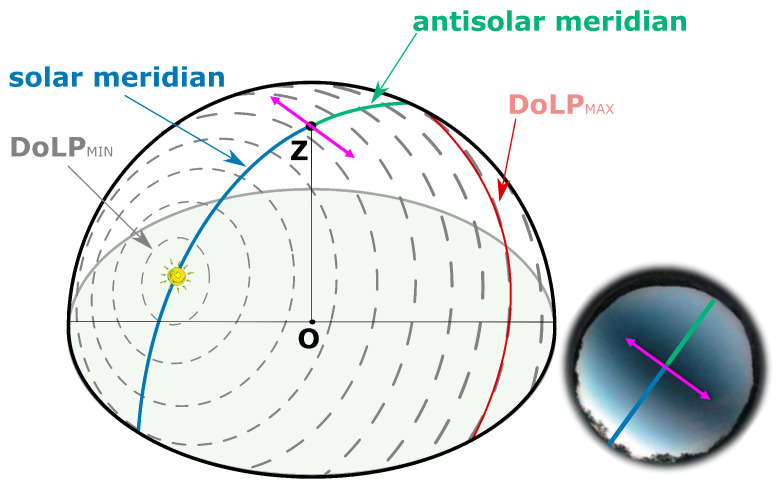
Polarization pattern of skylight as a function of the position of the sun and relative to an observer (O). The point (Z) represents the zenith. The light green horizontal disc is considered tangent to the Earth’s surface; the O–Z axis is taken as the normal to this plane. The orientation of the black dashes gives the direction of polarization, while the thickness describes the Degree of Linear Polarization (DoLP). The direction of polarization is orthogonal to the solar and anti-solar meridians (pink double arrow). **Insert**. Photograph taken with a linear polarizing filter under a clear sky. By orienting the polarizing filter to the same direction as the solar meridian (blue line), you can see a darker bar (double pink arrow) perpendicular to the solar meridian.

Whatever the position of the sun in the sky or below the horizon, the pattern of angles or degrees of polarization is symmetrical with respect to the solar and anti-solar meridians [190] which are formed by the semicircle passing through the zenith and the sun (Figure 6). It is precisely these symmetries that have led to the use of polarization as a form of heading information by navigating insects [13].

**Figure 7:**
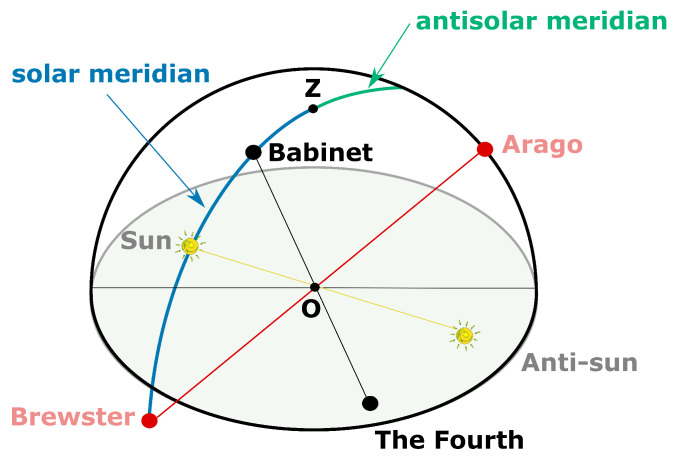
The polarization pattern of the Berry model does not correspond to the Rayleigh model and breaks its circular symmetry (see Figure 6) by introducing four neutral points. These four neutral points are named Brewster (below the sun), Babinet (above the sun), Arago (above the anti-sun) and the Fourth (below the anti-sun). However, the solar–antisolar meridian symmetry remains in the polarization pattern. The points (0) and (Z) represent, respectively, an observer and the zenith.

The skylight’s polarization pattern can be either simulated by using the Berry model [191,192,193] or a more complex approach based on Mie scattering and Monte Carlo simulations [194]. The limitation of the Rayleigh model is due to atmospheric turbidity and multiple scattering. Indeed, the global polarization pattern does not correspond to the Rayleigh model and we can actually observe four points featuring null DoLP [195,196,197,198], which are called neutral points. As shown in Figure 7, these four neutral points are named Brewster (below the sun), Babinet (above the sun), Arago (above the anti-sun), and the Fourth (below the anti-sun). They are either located on the solar meridian or the antisolar meridian (Figure 7); however, for a fixed sun position, their respective elevations vary with the level of atmospheric turbidity and the wavelength of light [196,197].

The Berry model, more accurate than the Rayleigh model, can be useful for better heading measurements [199,200] relying on neutral points detection [201] but cannot be fully used as a pattern prediction model because the positions of neutral points are strongly modified by air pollution, clouds, and debris from large volcanic eruptions [187,202], and because it lacks accuracy near the sun and the horizon. A recent study [203], considered the influence of the solar altitude angle on the neutral point position variations to model the pattern of polarized skylight. This model greatly improved the similarity between simulation and measurement data, but it was only developed in clear sky conditions and short periods of time (a couple of hours) [203].

### 3.3. Polarization-Based Sensors Dedicated to Navigation

**Figure 8:**
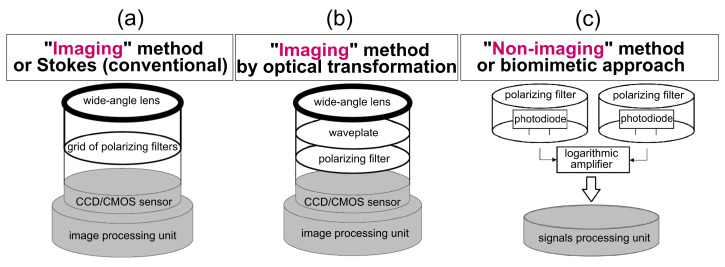
State-of-the-art polarimetric compasses. (**a**) Imaging method or Stokes (conventional). (**b**) Imaging method by optical transformation by mean of a waveplate (S-waveplate or linear waveplate). (**c**) Non-imaging method or biomimetic approach by mean of a set of photoreceptors, each one covered by a polarizing filter.

There are three main families of “polarimetric” heading detection techniques: imaging method or Stokes (conventional, see Figure 8a), imaging method by optical transformation by mean of a waveplate (S-waveplate or linear waveplate, Figure 8b) , and non-imaging method or biomimetic approach by mean of a set of photoreceptors, each one covered by a polarizing filter (Figure 8c).

#### Terrestrial Robots

**Figure 9:**
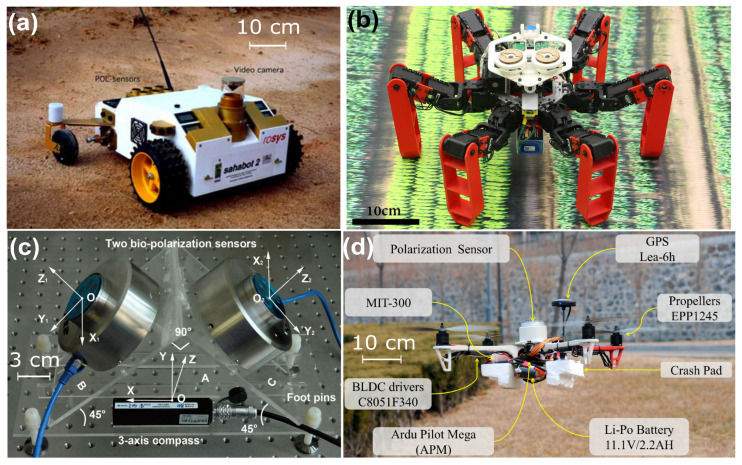
(**a**) The Sahabot 2 robot (2000) with its ant-inspired compass from Ref. [10] with permission of Elsevier. (**b**) AntBot robot equipped with a pair of UV-polarized light sensors forming a celestial compass from Refs. [11,12]. Photographic credits: Julien Dupeyroux, The Institute of Movement Sciences, CNRS/Aix Marseille Université, 2019. (**c**) Device based on two polarization sensors measuring the heading from Ref. [207] under CC-BY License, 2015. (**d**) Implementation of an extended Kalman filter on board a quadrotor for incorporating the polarization sensor into a conventional attitude determination system, from Ref. [208] under CC-BY license, 2018.

Insects possess photoreceptors in the dorsal region of their eye (the Dorsal Rim Area, DRA) that are specialized in detecting the pattern of polarized skylight [204]. The first robotic application of the desert ants’ DRA was implemented on board a mobile robot, called Sahabot in 1997 [205] and again in 2000 [10] (Figure 9a). The first robust ant-inspired celestial compass composed of only two photodiodes covered with rotating polarized filters (Figure 8c) was implemented on board a hexapod robot, called AntBot [11,12,206] (Figure 9b). The median AoLP error of the AntBot method was 0.4° under clear sky and 0.6° in the case of overcast weather [206].

#### Aerial Robots

A pair of polarized-light sensors based on a group of six photoreceptors, each photoreceptor being covered by a piece of polarizing filter, was tested under a clear sky (Figure 9c) and led a heading accuracy of 0.2° [207]. A single unit, shown in Figure 9c, was mounted on board a quadrotor (Figure 9d), providing an outdoor heading accuracy better than 2° with an output refresh signal of 10 Hz [208].

Yang et al. took inspiration from insects’ DRA to design and manufacture a POL-unit (a pair of photosensors covered with a pair of orthogonal polarized filters, see Section 3.3.3 for further information) based on a polarizing beam splitter (PBS) in order to avoid quadrature error of polarizing filters [170]. Each POL-unit is 5.5 × 5.5 × 6.5 cm in size and 50 g in weight and possess a heading accuracy of 0.12º with an output refresh signal of 10 Hz [170]. Three POL-units were mounted on board a 6 kg six-rotor Unmanned Aerial Vehicle (UAV) [209]. In static experiments, they reached a three-dimensional accuracy of less than 0.2°, and in dynamic experiments, they reached three-dimensional accuracy of 2.9° in pitch, and 1.9° in yaw and roll [209]. Adding a Polarization-based Compass (PbC) in an integrated navigation system is relevant to better estimate the attitude in flight. In dynamic experiments on board a six-rotor UAV, Qui et al. demonstrated that the estimation error of the integrated navigation system could reach a value as low as 0.3° around each axis [210].

A polarized camera (based on Sony sensor IMX250MZR, 2448 × 2048 pixels, 24 fps, such as mentioned in Section 2.2.5) was mounted on board a 15 kg six-rotor UAV [211]. Polarimetric images were processed by gated recurrent unit (GRU) neural network generating an output refresh signal of 10 Hz with a heading accuracy of 0.5°. The dynamic experiment was performed at an altitude of 310 m over a flight distance of 500 m [211].

Non-imaging and imaging-based PbCs are therefore relevant for both heading and attitude measurements in outdoor navigation [212]. PbCs are useful to work in GNSS-denied or magnetic disturbance environments in which the sky dome is still visible. PbCs can therefore significantly improve both heading and attitude measurements by fusing them with inertial sensors [211].

#### Military Devices

**Figure 10:**
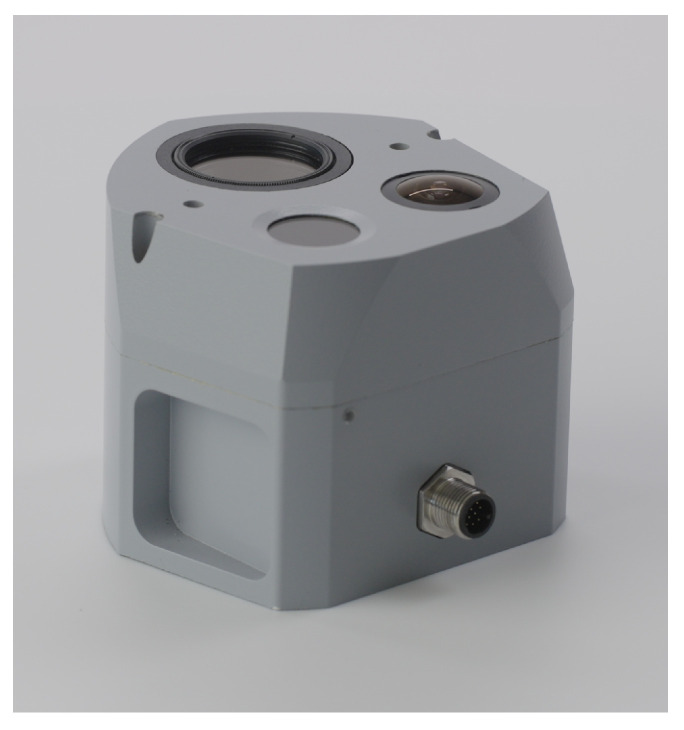
The SkyPASS Gen3-N sensor (size: 10.4×9.9×8.1 cm, mass: 567 g, max measurement frequency: 1 Hz) employs separate optical channels to image the sun, stars, and sky polarization to provide a highly accurate heading better than 0.1°. Tracking sky polarization improves availability of the sensor in twilight, cloudy skies, and urban environments. Courtesy from Polaris Sensor Technologies Inc. (Huntsville, AL, USA) , see https://www.polarissensor.com/skypass/ (accessed on 19 March 2024) for details.

SkyPASS is a unique military device available on the US market (cost in excess of USD 10k for production units, Figure 10) [159,160,161,162] which claims an accuracy compatible with fielded military systems (heading accuracy better than 0.1° in static experiments (see https://www.polarissensor.com/skypass/, accessed on 19 March 2024) and 0.5° in dynamic experiments [162]). The SkyPASS polarization-based celestial compass algorithm is based on pattern matching imagery collected with simulated information and data fusion with GNSS signals. To date, no civilian polarization-based compasses are on the market. DoFP polarization cameras are available with a unit cost of USD 1.5k–3k; however, this price is too expensive for service robotics or mass-market automotive applications. All other civilian research studies in China claim a heading accuracy in the range 0.2°–1° [168,169,170,171,172,173,174,175,176,177,178,179,180,181,182].

Field experiments with a polarimetric camera (BFS-U3-51S5P-C, FLIR, based on Sony sensor IMX250MZR, FLIR Systems Inc., Wilsonville, OR, USA) equipped with a 185° fisheye lens (FE185C57HA-1, Fujinon, Fujinon Corporation, Saitama, Japan) achieved real-time, robust, and accurate performance under different weather conditions with a Root Mean Squared Error (RMSE) of 0.1° under a clear sky, 0.18° under an overcast sky with a thin layer of clouds, and 0.32° under an isolated thick cloud cover [213]. This level of accuracy is relevant for military applications, where the bearing of true north must be detected with an accuracy of less than 0.1°.

#### Automotive Applications

Celestial compasses were also embedded on the top of automobiles to estimate their headings in dynamic experiments, with an RMSE of 0.81° [214] around a park, an RMSE of 0.55° in [164] along a straight boulevard, and an RMSE of 1.86° in [165] in an urban environment. Of course, dynamic experiments are more relevant for robotic applications rather than static experiments, and the heading accuracy is strongly affected by movements in dynamic experiments (e.g., an RMSE of 0.28° in static experiment *versus* an RMSE of 0.81° in dynamic experiments in [214]).

#### Ant-Inspired Path Integration

Several robots have been fitted with a PbC in order to implement an ant-inspired path integration [215]. Originally, the mobile robot Sahabot 2 (Figure 9a) suffered from a homing error as small as 0.2% (ratio homing error-traveled distance) along a traveled distance of 70 m in the desert [10]; the hexapod robot AntBot (Figure 9b) had an error as small as 0.7% along a traveled distance of 7 m over a flat terrain; and the mobile robot Turtlebot2 had an error of around 1.1% along a road of 45 m [216]. Zhou et al. also tested its PbC/INS along a traveled distance of 125 m comprising 14 checkpoints and reached a positioning error along the trajectory of approximately 0.5% [216], which represents the longest traveled distance of all ant-inspired robots, as far as we know. All these results need to be confirmed over longer distances and in various environments but adding a PbC in an integrated navigation system is always beneficial and can improve the heading and trajectory accuracy by about 40% [22].

The low level of positioning error is certainly correlated to the output refresh rate of the PbC, which was at 1 Hz with the Turtlebot2 [216], barely at 0.05 Hz on board AntBot [11,12,206] (Figure 9b), and an analog output with the Sahabot 2 (no information found in [10]. However, non-imaging and imaging-based PbCs can now reach 10 Hz [170,209,211], which could be relevant in the next decade to evaluate the ant-inspired path integration over distances of several hundred meters and observe whether the positioning error remains bounded in the 0.2–0.5% range.

#### 3.3.1. Celestial Compasses Based on Stokes Methods

When designing a pixelated polarized light compass based on the Stokes’ formalism (see Figure 8a and Section 2.1), it is crucial to take into account the various errors of the sensors like biases, gains and mechanical errors (also called installation error) due to alignment errors between the CCD pixel array and the micropolarizer array [217]. These errors are also considered for non-imaging sensors such as a compound eye polarization compass [218]. As a consequence, calibration efficient methods have been developed to compensate for the various errors [84,89,111,112,166,219,220]. The orientation of the camera in the inertial frame also called camera model can be determined by means of an inertial measurement unit or from solar observation for a camera equipped with a fish-eye lens [221,222,223]. A standard method used to estimate the heading relies on the fact that E-vectors are perpendicular to the sun vector leading to the calculation of eigenvalues [217,224]. Other studies exploit the symmetry pattern of the AoLP [172], also called the *∞* characteristic model [225]. Similarly, a few studies have applied the Hough transform to the AoLP pattern [200,226].

#### 3.3.2. Celestial Compasses Based on Imaging Methods by Optical Transformation

In 2016, Zhang et al. proposed an unexplored method based on a photosensor coupled to a radial polarizer [227]. This method can be classified as an aperture-coded light field capture method. As shown in Figure 8b, the grid of linear polarizing filters is replaced here by a “S-waveplate” and a linear polarizing filter acting as a radial polarization converter. However, the choice of a Raytrix-R29 light field camera limited the maximum frame rate at 1Hz, which is not relevant for mobile robots with fast dynamics even if they could reach a great accuracy (<0.2°) with a 300×300 pixels images resolution [227].

The PbC referred to in [227] uses a variation of an optical component called an S-waveplate which has spatially variable properties; depending on the zone where the light ray penetrates, the birefringence and the slow/fast axes will not necessarily be identical. Such a waveplate has the property of transforming a ray with a homogeneous distribution of polarization state into another polarization state (in particular, it transforms a spatially and linearly polarized homogeneous ray into a radially polarized ray). The PbC designed in Zhang et al. [227] is therefore not based on the variation in incidence of the rays of a spatially homogeneous component, but on the variation in the spatial properties of a component different from the one, called here a “S-waveplate”. A conventional “waveplate” (also known as a retarder plate) consists of a spatially homogeneous optical material with a certain amount of birefringence, which affects the state of the incident polarization in the same way as two parallel rays passing through the plate along different paths. In 1944, Bernard Lyot theorized the dependence of retardance on the incidence of rays in the case of a homogenous birefringent material, in order to develop his own wavelength polarizing filter [228]. Zhang et al. [227] only tested their S-waveplate on skies with a fairly homogeneous luminosity and an overall high DoLP (basically blue skies with no sun in the field of vision or clouds).

**Figure 11:**
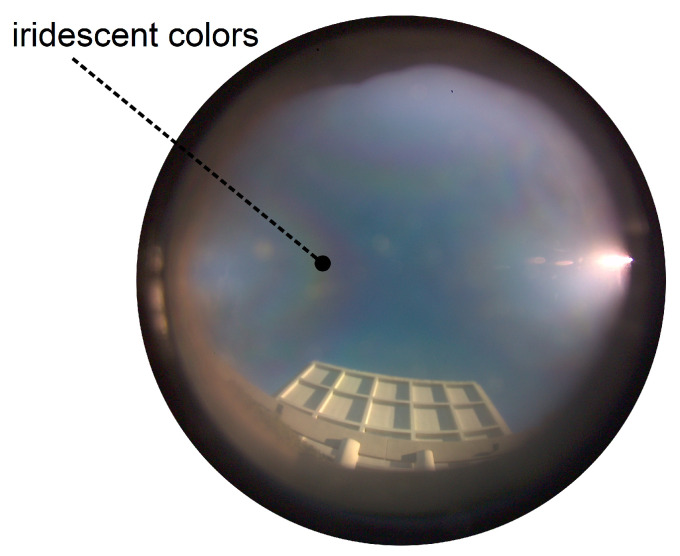
PILONE view. Outdoor image acquired by a Raspberry Pi color camera and obtained by clear sky in front of a building. Iridescent colors can be seen. From [230] under CC-BY license, 2023.

Poughon et al. proposed another heading sensor architecture based on polarization pattern estimation using a conventional waveplate [229,230] (see Figure 8b). This optical architecture, called PILONE, is based on variations in the retardance of a waveplate as a function of the angle of incidence of the polarized light rays, resulting in the appearance on the image of iridescent colors depending on the orientation of the incident rays and the state of polarization (Figure 11). The estimation of sun orientation from clear sky images with sun hidden artificially is based on a convolutional neural network [229]. Working with a Raspberry Pi color camera capable of 30 fps frame rate, this architecture may be relevant for real-time mobile robotics, with preliminary results showing a clear-sky accuracy in the 1° range with 64×64 pixels undersampled images [229,230]. The PILONE PbC results in a low-cost lightweight sensor that would cost about the same as the color camera used (here a Raspberry Pi wide angle camera, i.e., a few dozen euros), which may be relevant for applications in both automotive and robots manufacturing.

#### 3.3.3. Celestial Compasses Based on Non-Imaging Methods or Biomimetic Approaches

Non-imaging methods for implementing a PbC (Figure 8c) can be sorted into two main categories. The first relies on the Malus law [231], which gives the output signal $S_{i}$ of a pixel combined with a polarized filter defined as follows:

(10) $$S_{i}=K\cdot I\cdot \left[1+d\cdot \cos\left(2\Phi -2\phi_{i}\right)\right]$$

where *K* is the pixel gain, *I* is the incident light intensity, Φ is the polarization azimuth of the compass, $\phi_{i}$ is the theoretical orientation of the polarized filter of the *i*-th channel, and *d* is the ratio of the intensity of fully polarized light to the total light intensity. The Equation (10) can be written in a matrix form to estimate the various parameters *K*, *d*, and *I* from the measurements $S_{i}$ by a applying a standard non-linear least-squared method. The second category concerns method based on the so-called Labhart’s model of the POL neuron in crickets, the frequency of which is a sinusoidal function of e-vector orientation [204,232,233,234]. As depicted by [204], a POL-unit implements the log ratio of two photosensors *S* with two orthogonal polarized filters (here $\phi_{1}=0^{\circ }$ and $\phi_{2}=90^{\circ }$):

(11) $$p_{1}\left(\phi \right)=\log\left(\frac{S_{1}}{S_{2}}\right)=\log\left(\frac{1+d\cdot \cos(2\Phi )}{1-d\cdot \cos(2\Phi )}\right)$$

**Figure 12:**
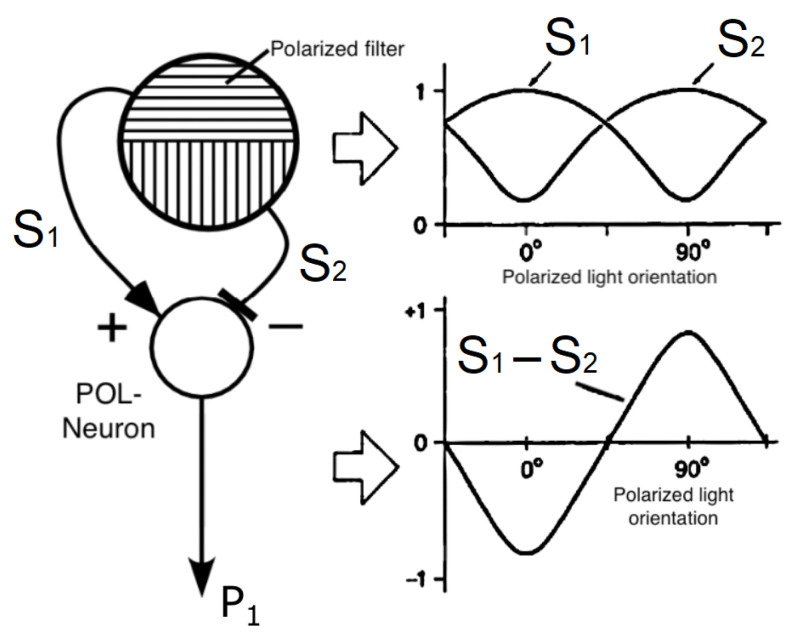
Model of a POL unit that accounts for the e-vector response of two crickets’ photoreceptors endowed with their orthogonal polarized filter (noted here as 1 and 2). The POL neuron (output signal $p_{1}$, see Equation (11)) performs the log ratio of the two photoreceptors’ output signal ($S_{1}$ and $S_{2}$). Adapted with permission of the Journal of Experimental Biology [234].

As depicted in Ref. [235], the AoLP and DoLP can be calculated from Equation (11) by means of an analog logarithm amplifier [171] and by orienting several POL units (Figure 12) along various orientations. The logarithm amplification gives the ability to deal with a large range of lighting conditions over several decades. Thus, it becomes possible to make an array of POL units distributed on a planar surface [236] along a circular [167,237,238] or even spherical shape mimicking a compound eye [239,240,241]. Moreover, a spherical sensor composed of several POL units was used to compensate for the error alignments of an inertial measurement unit on the basis of sun and star vectors [241].

### 3.4. Polarization-Based Geolocalization

#### 3.4.1. Polarization-Based Geolocalization Using Solar Ephemeris

Yang et al. [180] and Zhang et al. [182] proposed a polarization-based geolocation method relying on the maximum degree of polarization required to find the sun position with an artificial compound eye comprising 54 photodetectors calculating a coarse geographic position (latitude error: 0.11°, longitude error: 0.08°, spatial error: hundreds of kilometers). Powell et al. [242] proposed an underwater polarization-based geolocation method using an imaging method and reached a spatial error of about 100 km.

As far as we know, only PbCs have been combined with Integrated Navigation Systems (INS). They could be either based on a PbC/INS with a heading accuracy ranging from 0.08° to 0.8° in sunny weather, but 0.8° to 1.5° in cloudy conditions [179,240,241,243,244,245,246], or PbC/GNSS/INS with a heading accuracy ranging from 0.02° to 6.5° [175,247,248,249], or PbC/SLAM/INS with a heading accuracy from 0.28° to 4.7° and a geographical position error from 1.96 m to 8.7 m [214,250,251,252,253]. The heading and trajectory accuracy can therefore be improved by about 40% compared to conventional navigation systems in complex outdoor environments [22].

#### 3.4.2. Polarization-Based Geolocation Using the North Celestial Pole (SkyPole Algorithm)

**Figure 13:**
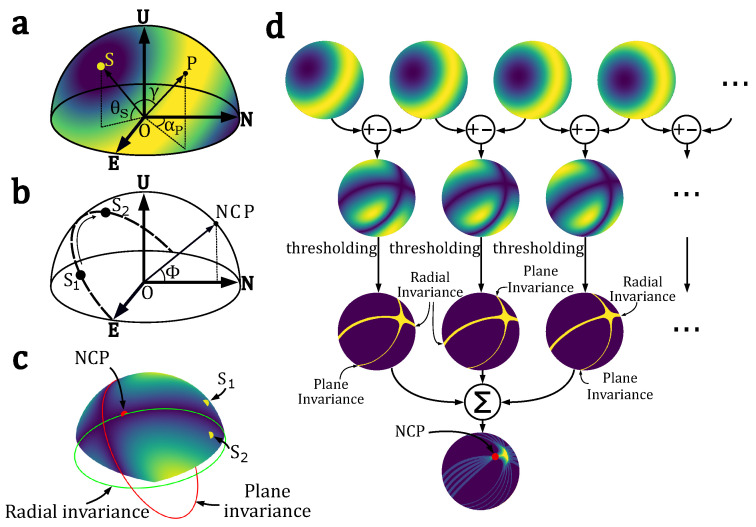
(**a**) Scattering angle γ, azimuth $\alpha_{P}$ of a point P, and solar altitude $\theta_{S}$ of the sun S. The parameters are depicted in the ENU (East, North, Up) coordinate system centered on the observer O. The color pattern represents the Degree of Linear Polarization (DoLP) in the sky, described by the Rayleigh single scattering model [187]. Dark blue represents a near-zero DoLP and yellow represents maximum DoLP values. (**b**) Sun trajectory in the ENU coordinate system, centered on observer O, positioned at latitude ϕ. The sun moves in a plane perpendicular to the observer-NCP vector. (**c**) DoLP invariances on the celestial sphere. Invariance circles are computed from analytical calculus. The colored half sphere is the simulated absolute difference of two DoLP patterns linked to the sun’s positions $S_{1}$ and $S_{2}$ at two distinct times. Dark blue represents near zero values. (**d**) Method for finding the NCP from the sky’s DoLP pattern. The first row displays DoLP patterns taken at four different moments. The absolute differences between the DoLP patterns are then computed and shown in the second row. Thresholding is applied to these images (third row). Finally, the binary images are overlaid, and the NCP is located at the intersection of radial invariance axes. From [254] under CC-BY-SA-ND license, 2023.

The use of solar ephemeris combined with an estimation of the ’s position through the polarization pattern enables direct geographical positioning [180]. Nevertheless, animals do not have access to these ephemerides (Figure 13b), and the utilization of the polarization pattern (Figure 13a) as a reference for their navigation remains poorly understood. In 2023, an alternative method inspired by migratory birds was proposed [254]. Migratory birds calibrate their magnetic compass through the celestial rotation of night stars or the daytime polarization pattern [3,255]. Similar to Brines [256], the temporal properties of the sky’s polarization pattern were considered as relevant navigation information. For this purpose, a bio-inspired method to find geographical north and the observer’s latitude was developed [254], requiring only skylight polarization observations provided here by a commercial polarimetric camera. Skylight is mostly linearly polarized and characterized by two parameters: AoLP and DoLP. This method consists of processing only skylight DoLP images taken at different instants in order to find the north celestial pole (NCP) from temporal invariances of the DoLP pattern (Figure 13c,d). Then, the geographical north bearing (true north) and the observer’s latitude Φ (Figure 13b) can be deduced from the NCP’s coordinates.

To experimentally validate the NCP approach, a polarimetric camera (PHX050S-QC from Lucid Vision Labs, sensor ref. Sony IMX250MYR) equipped with a 185° fisheye lens (FE185C57HA-1, Fujinon) was used and situated on the roof of the Institut de Neurosciences de la Timone (INT), Marseille, France (43.2870135° N, 5.4034385° E). The study yielded a Mean Absolute Error (MAE) of 2.6° in azimuth and 3.8° in latitude [254].

#### 3.4.3. Can the Underwater Sky Polarization Be Useful for Navigation Purposes?

In 1954, Waterman demonstrated that polarized light from the sky was accessible under clear water at depths of up to several hundred meters, using many behavioral observations of underwater animals in connection with their migration mechanisms [257,258]. When observed from the calm surface of the water looking upward, the perspective above the water’s surface becomes condensed into a conical angle of 97.5° due to the refraction effect. This underwater field of view is commonly referred to as Snell’s window [259]. The underwater model of polarization patterns available in calm seas is well established; however, few studies have modeled them with waves [260]. Although the ability of certain animals to use underwater polarization as a compass for navigation is still under debate, it could be worth studying the properties of underwater polarization. It has been clearly shown that the degree of polarization is stable and consistent with the sun’s location at depths of 2 and 5 m only in clear waters [261]. However, the influence of water turbidity on the refraction-polarization pattern can probably be ignored within the topmost thin surface layer of seawater, where polarization vision of aquatic animals is located in the UV range [262]. In their review, Cronin and Marshall recalled that the polarization pattern is strongly affected by the depth from which the pattern is measured [263]. These vertical variations depend on the amount and quality of suspended material in the water. As depth increases, multi-path scattering destroys the pattern coming from the sky and only in-water scattering, produced near the observer, remains. At very low depth, the influence of wavy water surfaces on the polarization pattern has been simulated and measured [264]. It was revealed that the wind speed also has an influence on the pattern. Powell et al. proposed estimating the position of an observer by processing underwater polarization patterns with a custom-made polarimetric camera [242]. Geolocalization was achieved here by means of accurate knowledge of time and date. The accuracy obtained was a 6 m error for every 1km traveled at depths ranging from 2 m to 20 m. A recent study based on deep learning reached geolocation accuracies of 55 km at a depth of 8m and 255 km at a depth of 50 m even in low-visibility waters [265]. Accurate heading estimation of an autonomous underwater vehicle was recently obtained by merging inertial and polarization information [266,267]. A standard deviation (SD) error of 0.83° was reached here at a depth of 2 m in real oceans in calm seas [267]. Cheng et al. confirmed the deterioration of the heading measurement as a function of depth from 0.93° at a depth of 1 m to 4.07° at a depth of 5 m [266].

### 3.5. Summary and Future Directions in Polarized Vision for Robotics Navigation

Combining Strapdown Inertial Navigation Systems (SINSs) or Inertial Measurement Unit (IMUs) with polarized sensors is of great interest for improving the dynamics and accuracy of the estimated variables of interest (pitch, roll, yaw, positions, etc.). For example, heading estimation has been considerably improved by merging SINS with a spherical polarized sensor [240]. In addition, it has been shown that a method based on spherical non-imaging polarimetric sensor composed of nine POL units was able to estimate the static position of the sensor with a positioning error as small as 0.07° in latitude and 0.012° in longitude [239]. Finally, an autonomous robot was able to home with a position error twice as small as a method only based on IMU [216].

Challenges remain. By and large, skylight polarization in the UV range has seldom been exploited for robotic applications, none of the commercial or experimental devices detect the surrounding light in a full panoramic view as insects do, and none of them can work in cloudy or extreme weather conditions [268].

Most available polarized sensors are megapixel cameras, which are bulky and expensive for applications in automotive or service robotics, in which low cost will be a prerequisite (<USD 1000). In terms of learning from insects, the desert ant *Cataglyphis* is able to detect its celestial heading by using only 100 ommatidia in their DRA in each compound eye comprising 1300 ommatidia, each ommatidium in the DRA comprising six UV-sensitive photoreceptors. As a result, the number of required photoreceptors to detect the celestial heading in the same manner as desert ants *Cataglyphis* [2,269] is 1200 UV-sensitive photoreceptors. This number of photoreceptors is equivalent to a 34×34 pixels thumbnail image, which corresponds to an intermediate resolution between non-imaging and imaging sensors. Thumbnail images from 22×22 pixels to 64×64 pixels have been already used to train and validate neural networks [229,230]. Neural networks are promising solutions in robotics to process thumbnail images in real time, but they will require the design and manufacture of dedicated artificial retinas comprising approximately one thousand pixels instead of millions of pixels.

Generating neural networks in order to detect attributes of the polarization pattern, or denoise or interpolate polarization patterns will require image databases, either generated by simulation, or acquired by polarimetric cameras [26,270]. These images databases are now available and will be use either to train or to validate neural networks [223,271] which will be relevant in robotics for processing polarimetric images in real time. Optical transform-based imaging methods will be inexpensive but will require a bank of images for calibration.

Non-imaging methods require a grid of integrated polarizing micro filters, which will become cheaper as production methods focused on heading detection improve.

The development of miniaturized and all-day sensors will be compatible in terms of both size and cost with service robotics. These sensors’ outputs will be merged into an integrated navigation system as a supplemental perceptive modality of localization techniques to complement and reinforce the conventional ones. Polarization patterns can also be used at night with the moon in the same manner as ants [272]. It has already been proven that we can reach a heading accuracy of 2.45° at night with polarized light alone but under the same conditions and by combining all the moon’s light pattern properties, we can reach an accuracy up to 0.5° [273]. All these experiments were carried out during full moons in favorable environments. Conducting such experiments in unfavorable conditions remains a big challenge. The moon’s polarization pattern can also be used for positioning at nighttime. Chen et al. obtained a positioning error within tens of kilometers, yielding a latitude accuracy of 0.62° (1σ) and a longitude accuracy of 0.02° (1σ) [274]. Yet, how all light patterns properties (polarization information and intensity information) of the sun or the moon could be combined to obtain the best level of performance remains an open question.

## 4. Polarized Vision for Scene Understanding

In nature, light polarization occurs mainly due to two physical phenomena: light scattering and light reflection [275]. As an illustration of the latter property, many animal species such as water fleas and butterflies are sensitive to the polarization of light and exploit this ability to discriminate water [276]. This section focuses on how robots may take advantage of sensing polarized light to understand scenes through detection, estimation of 3D shapes, depth and pose estimation. Ref. [277] may be the first in the literature to emphasize, in the computer vision field, how the polarization parameters of light are related to the normal estimation of objects. In this section, after recalling and deriving all the mathematical formula linking the polarization parameters to the normal orientation, direct application, i.e., object detection and discrimination, will be described. Shapes from polarization that exploit most of the physical information will be then presentedand the section will end with the latest techniques using polarization imaging to improve depth estimation and or facilitate pose estimation in robots.

### 4.1. Polarization and Reflection

The reflection model employed here is a simplified one, providing a first approximation of the use of polarimetric imaging for the detection and 3D reconstruction of objects. In practice, reflected light is a combination of these two reflection: diffuse and specular. Reaching an interface between two media with different properties, light becomes partially reflected and partially transmitted. Considering a beam traveling through the first medium (characterized by a refractive index $n_{1}$) then reaching the interface of a second medium (characterized by refractive index $n_{2}$), the proportions that are reflected and transmitted are defined by the Snell’s Law:

(12) $$\left\{\begin{array}{c} n_{1}\cdot \sin\theta_{i}=n_{2}\cdot \sin\theta_{t}\\ \theta_{r}=\theta_{i} \end{array}\right.,$$

where $\theta_{i}$, $\theta_{t}$, and $\theta_{r}$ are the angle of incidence, transmission, and reflection, respectively. In addition, the incident, transmitted, and reflected beams are in the same plane containing the normal of the surface and called the plane of incidence.

#### 4.1.1. Recall of Fresnel Formulae

The Fresnel formulae can be determined by solving Maxwell’s equations and respecting the continuity conditions imposed at the interface on the electric and magnetic fields. Letting *r* and *t* denote the reflectivity and transmissivity ratios that represent the ratio of the complex amplitude of the reflected and transmitted values with the amplitude of the electric vector of the incident field, we have the following (see [28]):

(13) $$\begin{array}{c} \left\{\begin{array}{cc} r_{\|}= & \frac{\tan\left(\theta_{i}-\theta_{t}\right)}{\tan\left(\theta_{i}+\theta_{t}\right)}\\ r_{⊥}= & -\frac{\sin\left(\theta_{i}-\theta_{t}\right)}{\sin\left(\theta_{i}+\theta_{t}\right)} \end{array}\right.\\ \left\{\begin{array}{cc} t_{\|}= & \frac{2\cos\theta_{i}\cdot \sin\theta_{t}}{\sin\left(\theta_{i}+\theta_{t}\right)\cdot \cos\left(\theta_{i}-\theta_{t}\right)}\\ t_{⊥}= & \frac{2\cos\theta_{i}\cdot \sin\theta_{t}}{\sin\left(\theta_{i}+\theta_{t}\right)} \end{array}\right. \end{array}$$

where ‖ (resp. ⊥) denotes the components in the plane of incidence (resp. normal to the plane of incidence).

#### 4.1.2. Partial Polarizer

The Mueller matrices of reflection and transmission are directly related to the Mueller matrix of a partial polarizer as defined in the following equation:

(14) $$M_{pp}=\frac{1}{2}\left[\begin{array}{cccc} {\left|a_{⊥}\right|}^{2}+{\left|a_{\|}\right|}^{2} & {\left|a_{⊥}\right|}^{2}-{\left|a_{\|}\right|}^{2} & 0 & 0\\ {\left|a_{⊥}\right|}^{2}-{\left|a_{\|}\right|}^{2} & {\left|a_{⊥}\right|}^{2}+{\left|a_{\|}\right|}^{2} & 0 & 0\\ 0 & 0 & 2ℜ\left\{a_{⊥}a_{\|}^{*}\right\} & 2ℑ\left\{a_{⊥}a_{\|}^{*}\right\}\\ 0 & 0 & -2ℑ\left\{a_{⊥}k_{\|}^{*}\right\} & 2ℜ\left\{a_{⊥}a_{\|}^{*}\right\} \end{array}\right],$$

where $a_{⊥}$ and $a_{\|}$ are the ratio coefficients according to the perpendicular and parallel of the incidence plane, respectively. Therefore, assuming the incoming light is unpolarized, this leads to obtaining light that is partially linearly polarized with a degree of polarization (DoP) equal to the following:

(15) $$DoP=\left|\frac{{\left|a_{⊥}\right|}^{2}-{\left|a_{\|}\right|}^{2}}{{\left|a_{⊥}\right|}^{2}+{\left|a_{\|}\right|}^{2}}\right|.$$

In addition, it can be deduced that the polarized vibrations are orthogonal to the plane of incidence if ${\left|a_{⊥}\right|}^{2}>{\left|a_{\|}\right|}^{2}$ and parallel otherwise.

#### 4.1.3. Specular Reflections

To study the polarization properties of the light that is specularly reflected, Equation (15) can be used by replacing $a_{⊥}$ and $a_{\|}$ by the Fresnel ratio of the reflection $r_{⊥}$ and $r_{\|}$ given from Equation (13). If we denote θ as the angle of reflection, denote *n* as the real refractive index of the media that the beam is reflected within, and assume that the refractive index of air is equal to 1, $\theta_{i}$ and $\theta_{t}$ can be rewritten:

(16) $$\left\{\begin{array}{c} \theta_{i}=\theta \\ \theta_{t}=\arcsin\left(\frac{\sin\theta }{n}\right) \end{array}\right..$$

Consequently, the Equation (15) of the degree of polarization can be rewritten as a function of the angle of reflection θ and the refractive index *n* [278]:

(17) $$DoP=\frac{2\sin\theta \cdot \tan\theta \cdot \sqrt{n^{2}-\sin^{2}}\theta }{n^{2}-2\sin^{2}\theta +\tan^{2}\theta }.$$

**Figure 14:**
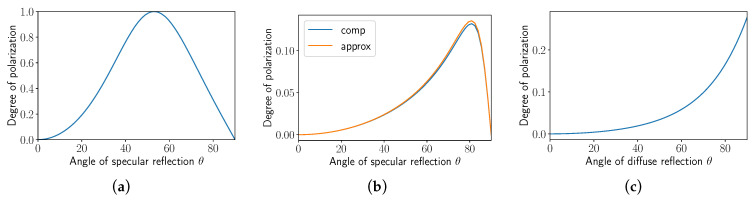
Relationship between the DoP (Degree of Polarization) and the reflected angle θ according to (**a**) dielectric specular reflection, (**b**) metallic specular reflection and (**c**) diffuse dielectric reflection.

Figure 14a shows the plot of the previous equation with a refractive index *n* set to 1.5. As can be highlighted here, an ambiguity occurs while trying to determine the θ angle from the DoP. The previous formulation of the DoP is only valid for dielectric materials. To derive a formula for a metallic object, the complex refractive index of the media $\hat{n}=n\left(1+i\kappa \right)$, where κ is the attenuation index, must be taken into account [279]. The following approximation can be applied if we consider the visible region of the spectrum of light [231]:

(18) $${\left|\hat{n}\right|}^{2}=n^{2}\cdot \left(1+\kappa^{2}\right)\gg 1.$$

Applying the same considerations as for dielectric objects, Equation (15) of the degree of polarization can be rewritten as the following:

(19) $$DoP=\frac{2n\cdot \tan\theta \cdot \sin\theta }{\tan^{2}\theta \cdot \sin^{2}\theta +{\left|\hat{n}\right|}^{2}}.$$

The plot presented in Figure 14b assumes a metallic medium and again reveals an ambiguity in the determination of angle θ from the measured DoP. Nevertheless, contrary to a dielectric object, the maximum occurs for a high value of the angle θ, around 80°, and the reconstruction the shape of smoothly curved objects can be applied without solving this ambiguity.

**Figure 15:**
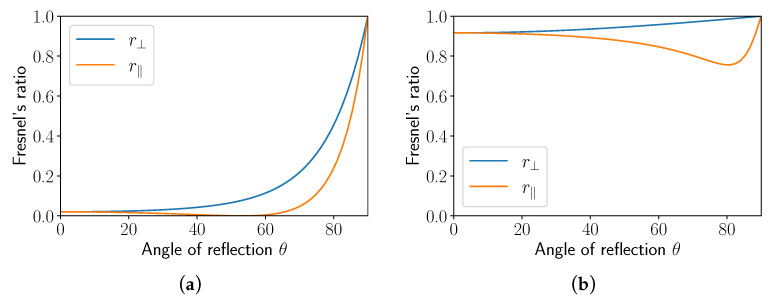
Fresnel’s ratio for specular reflection according to the angle of reflection: (**a**) dielectric object with refractive index equal to 1.33 , (**b**) metallic object with refractive index equal to 0.82+5.99j.

In addition, as can be seen in Figure 15, the orthogonal Fresnel ratio ${\left|r_{⊥}\right|}^{2}$ is always greater than the parallel one ${\left|r_{\|}\right|}^{2}$ in both cases: dielectric and metallic media. Therefore, we can deduce that the specularly reflected light becomes polarized orthogonaly to the incidence plane. As a result, polarization contrast that could be measured for materials with both types of reflection tends to reduce. Active polarization imaging, which is outside the scope of this review article, could be used to improve the contrast of such objects.

#### 4.1.4. Diffuse Reflections

The diffuse reflections that can provide polarized light are generally considered as the resulting process of light that first penetrates the surface and becomes partially polarized by refraction. Then, within the medium the light is randomly scattered and becomes depolarized. Some part of the light is then refracted back into air and becomes polarized. To obtain an expression of the degree of polarization according to the angle of diffuse reflection, Equation (15) can be used by replacing $a_{⊥}$ and $a_{\|}$ by the Fresnel ratio of the transmission $t_{⊥}$ and $t_{\|}$ given from Equation (13). With θ denoting the angle of diffuse reflection, *n* denoting the refractive index of the media that the beam is refracted from, and assuming that the refractive index of air is equal to 1, $\theta_{i}$ and $\theta_{t}$ can be rewritten:

(20) $$\left\{\begin{array}{c} \theta_{t}=\theta \\ \theta_{i}=\arcsin\left(\frac{\sin\theta }{n}\right) \end{array}\right..$$

The degree of polarization of the light in the case of diffuse reflections for dielectric objects can be rewritten:

(21) $$DoP=\frac{{\left(n-1/n\right)}^{2}\cdot \sin^{2}\theta }{2+2\cdot n^{2}-{\left(n+1/n\right)}^{2}\cdot \sin^{2}\theta +4\cdot \cos\theta \cdot \sqrt{n^{2}-\sin^{2}\theta }}.$$

Figure 14c shows the plot of the function linking the DoP to the angle θ. As can be seen, the DoP is lower in the case of a diffuse reflection than of a specular reflection. Nevertheless, the determination of the angle θ from the DoP is performed without any ambiguity if the refractive index *n* of the media is known.

**Figure 16:**
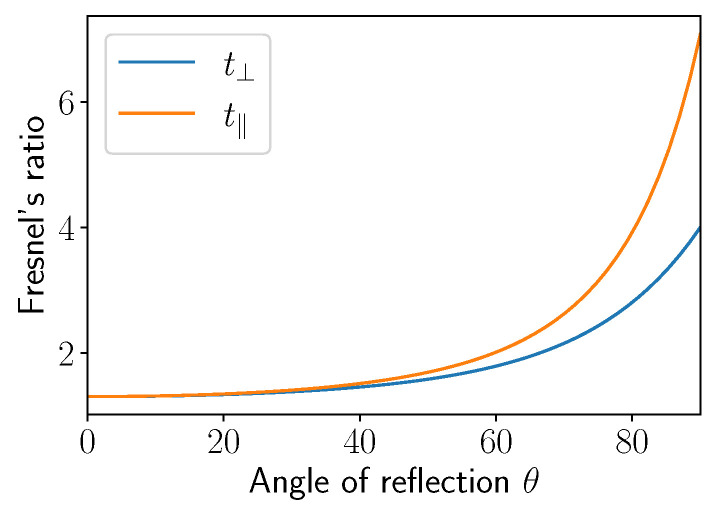
Fresnel ratio for diffuse reflection from a dielectric object according to the angle of reflection [231].

Also, contrary to specular reflections, as illustrated on Figure 16, the orthogonal Fresnel ratio is lower to that of the parallel one, which leads to the conclusion that light obtained by diffuse reflections is always parallel to the incidence plane.

### 4.2. Detection and Classification

Before finding some applications in robotics, detection and segmentation of objects based on polarimetric imaging were initially developed in the field of computer vision [277]. In Ref. [280], the physical basis was developed and detailed to highlight the capabilities of polarimetric imaging to distinguish metallic materials from dielectric materials. More advanced classification techniques can be found in [25]. Subsequently, the benefits of this modality to enhance perceiving of transparent objects were revealed [281]. This task is essential in robotic gripping systems to manipulate transparent objects with ease [282] and improvements are continuously being made [283].

**Figure 17:**
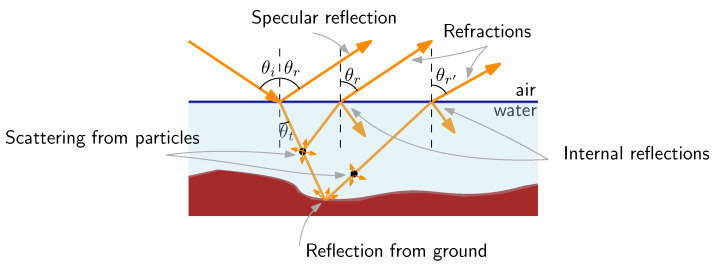
Reflection and refraction of light on water.

Autonomous robots are often based on bio-inspired systems regarding the perception task. Polarimetric cues are used by many water beetles and insects to search for bodies of water [276,284]. For instance, in ground robotics this modality has been exploited to detect water hazards or mud in conjunction with 3D sensing techniques such as LIDAR [285,286], stereo-vision [287,288], and mono-depth [289]. Figure 17 shows that the light reflected by water is made up of a proportion linked to the specular reflection as well as a proportion linked to refraction as described in the previous subsection. As shown in Figure 17, refraction is a combination of light scattered by particles in the water and light reflected by the ground. The Mueller matrix that models this phenomenon can be determined from the following:

(22) $$M_{water}=M_{refl}\left(\theta ,n\right)+\left(1-\mu_{absorption}\right)M_{refr}\left(\theta ,n\right)\cdot M_{dep}\cdot M_{refr}\left(\theta ,n\right)$$

where $M_{refl}$ and $M_{refr}$ are the Mueller matrices of reflection and refraction, respectively. $M_{dep}$ is the Mueller matrix of a depolarizer and $\mu_{absorption}$ is the absorption coefficient for both particles in the water and the ground. $M_{refl}$ and $M_{refr}$ can both be computed using the generic Mueller matrix $M_{pp}$ defined in Equation (14), replacing $\left(a_{⊥},\;a_{\|}\right)$ by the appropriate Fresnel coefficients defined in Equation (13): $\left(r_{⊥},\;r_{\|}\right)$ for the reflection case and $\left(t_{⊥},\;t_{\|}\right)$ for refraction. Using Equation (12) enables us to write the Mueller matrices as a function of the angle of reflection θ and the refractive index of water *n*.

Glass or transparent object segmentation remains a major issue in mobile robotics in urban environment to prevent collisions or misunderstanding of the scene. For instance, a learning-based method proposed in Ref. [290] that manages both polarization parameters and colorimetric information tends to outperform standard methods. More generally, the benefits of polarimetric imaging in urban scenes are still growing since it drastically improves the segmentation tasks. Among these, we can cite road classification [94,291,292,293], and semantic segmentation [294]. Advanced classification tasks can also be performed such as land mine detection [295,296] and astronomical solid body identification [297,298,299]. To increase the segmentation task quality, polarization modality can be used advantageously with infrared imaging [293,300,301] or multispectral imaging [296]. Reflection removal [302] can also be seen as a direct application of polarization properties of transparent surfaces to provide high-quality images for navigation tasks.

### 4.3. Shape from Polarization

**Figure 18:**
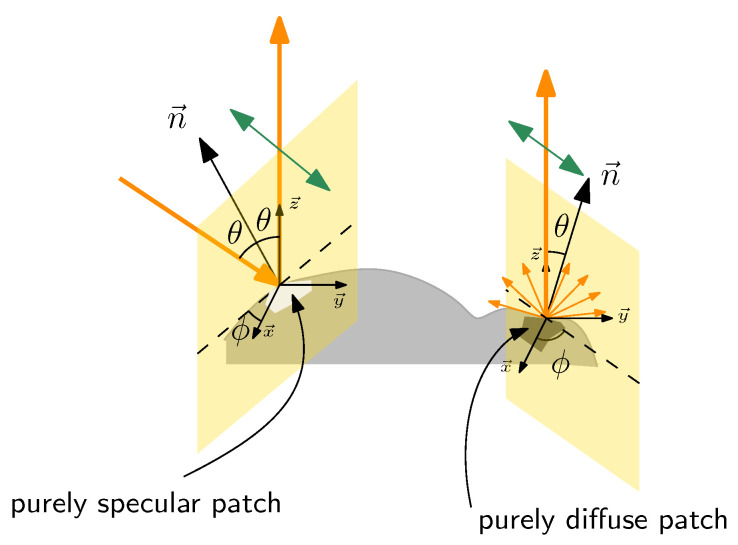
Illustration of the “Shape from Polarization” basis with the two types of reflection: specular and diffuse. The direction of polarization is indicated in green.

In most robotics tasks, the perception of three-dimensional objects, the estimation of depth, and 3D reconstruction are all essential. As presented in Section 4.1, the polarization parameters of the light reflected or refracted from an object are directly related to the normal of the surfaces. Historically introduced by Wolff and Boult [303], the way of determining the surface normals from the measured polarization parameters led to a specific field of computer vision named “Shape from Polarization”. Assuming as a first approach that an orthographic lens is used in front of the polarimetric sensor, all light rays are parallel according to the optical axis of the camera $\vec{z}$ as illustrated on Figure 18. In this frame, the normal can be written as the following:

(23) $$\vec{n}=\left[\begin{array}{c} \sin\theta \cdot \cos\Phi \\ \sin\theta \cdot \sin\Phi \\ \cos\theta \end{array}\right]$$

where θ and ϕ are the zenith and azimuth angles, respectively.

Finally the shape of the object is obtained by integrating the normal fields. The two angles θ and ϕ, respectively, are related to the degree and the angle of polarization. Depending on the nature of the surface, highly reflective or diffuse, some ambiguities appear in the determination of the normals:

**Diffuse reflection** As long as the refractive index is known, there is no ambiguity in determining the zenith angle θ from the DoP. The main drawback is that the DoP is lower for diffuse reflection. An ambiguity remains regarding the azimuth angle Φ which is equal to AoLP or AoLP+π, since the light is polarized in the plane defined by the normal and the reflected ray (Figure 18).
**Specular reflection** Assuming the refractive index is known, as shown in Figure 14, an ambiguity appears in the determination of the zenith angle θ from the DoP. In the same way, there is ambiguity as to the determination of the azimuth angle Φ which is equal to AoLP±π/2 since the light is polarized orthogonally to the incidence plane (Figure 18).

Shape from polarization started with the 3D reconstruction of objects having some priors about their shape to facilitate the disambiguation process [278,304,305,306]. Active lighting sources [279,307], multi-spectral imaging [308,309,310], multi-view [278] and Shape from Shading techniques [311,312,313,314] were also used in addition to shape from polarization to extract the most out of the techniques. Under flash illumination and using deep learning, Deschaintre et al. [315] captured the shapes of objects including the bidirectional reflectance distribution function by using polarization considerations. It is important to point out that multi-spectral imaging [309,310] in conjunction with polarization imaging enables the estimation of both the refractive index and the normals. Smith et al. [313] started with objects that provide both specular and diffuse reflections under controlled illumination, and later conducted experiments with unknown lighting [316]. To manage ambiguity between diffuse or specular dominating reflection [317] they were the first to introduce deep learning and to provide a lighting invariance algorithm based on shape from shading. Yang et al. [318] succeeded in using deep learning to reconstruct an object with shape from polarization information only. Knowing the polarization pattern of the blue sky [319] can also help to determine the object’s shape but this method is not suitable in real time. Evolution of shape from polarization is summarized in Table 2.

### 4.4. Three-Dimensional Depth with Polarization Cues

Thanks to its ability to estimate the normals, polarization imaging is increasingly involved in 3D depth estimation. To improve the 3D reconstruction of objects, Kadambi et al. [321] combine polarization imaging with an aligned depth map obtained from a Kinect. Disambiguation is initiated by the depth map and the integration process starts with depth map estimation and is then improved by including the estimation of normals.

#### 4.4.1. Stereo-Vision Systems

Also, in some stereo-vision systems, polarization imaging can solve the reconstruction of specular or transparent surfaces: Berger et al. [288] used a pair of polarimetric cameras to estimate the depth of a scene including the presence of water areas. Instead of using the polarization parameters to simplify the matching process, Fukao et al. [322] integrated all the measured parameters in a cost volume construction and the surface normals are then estimated. In a study carried out by Zhu and Smith [323], the pair comprises one RGB camera and one camera equipped with a linear polarizer (that could be replaced by a polarimetric camera). Even if restricted to controlled lighting, high- quality 3D reconstruction can be obtained combining polarization imaging and binocular stereo vision system thanks to the fusion scheme proposed by Tian et al. [324]. Cui et al. [325] proposed a multi-view acquisition system using polarization imaging that enables dense 3D reconstruction adapted for texture-less regions and non-Lambertian objects.

#### 4.4.2. Pose Estimation and SLAM

Pose estimation and SLAM (Simultaneous Localization and Mapping) are also of major importance in the field of robotics particularly for navigation tasks and scene analysis. Yang et al. [170] were the first authors to propose a polarimetric dense monocular that can reconstruct 3D in real time and provide improved results compared to conventional techniques when some regions are specular or texture-less.

Cui et al. [326] developed a relative pose estimation algorithm from polarimetric images which reduced the point correspondence to two points but limited the analysis of diffuse reflection. High reflections and transparency objects are handled in Ref. [327]. They developed a network called PPP-net (Pose Polarimetric Prediction Network) that uses a two step framework. The fusion of polarization information and physical cues provides after learning the object mask, normal map and NOCS (Normalized Object Coordinate Space) required to a final regression network for monocular 6D object pose estimation [328]. Additionally, a learning-based algorithm that focuses on human pose and shape estimation was recently developed by Zou et al. [329].

### 4.5. Summary and Future Directions in Polarized Vision for Scene Understanding

Polarization imaging is becoming an indispensable modality for robotics both as a means of providing additional clues regarding the nature of objects and as a major contributor to 3D object recognition. Nevertheless, as presented in this section, polarization imaging could not be a standalone system providing all necessary information. Ambiguities remain regarding the azimuth and zenith angles, or the priors of the refractive index or shapes, are sometimes unavoidable in robotics. Methods based on deep learning seem to overcome most of these limitations. Huang et al. [330] used a combination of stereo-vision and a polarization system to recover normals and disparity through a deep learning-based algorithm. Assuming only diffuse reflection, ambiguities were solved, and in addition, the authors succeeded in overcoming the restriction of using orthographic cameras. Consequently, standard stereo-vision systems can be advantageously replaced by a pair of color-polarized cameras.

Improved perception of the real world through polarization extends applications to more advanced systems such as event cameras [331,332] or iTOF (indirect Time Of Flight) cameras [333]. One solution to the major challenges in scene understanding and 3D estimation could be to fuse polarization cues through various wavelengths additionally to 3D sensor to provide robust reconstruction in the presence of specular or translucent objects that can be found indoors or outdoors. Extending the fusion of polarization imaging and multispectral imaging for the detection task appears to be relevant for scene understanding.

## 5. Conclusions

The principles of polarization that we use today have been known since the 19th century, but due to the lack of experimental imaging systems able to operate in real time, few applications were reported until the late 1990s, whatever the field. Availability of digital cameras and liquid crystal modulators made it possible to implement systems and a variety of applications such as skylight navigation was considered. Commercial systems emerged in the early 2010s due to a major increase in the availability of high-definition low-noise commercial cameras able to sense linear polarization at 50–100 fps.

The Division-of-Focal Plane (DoFP) camera for linear polarization image capture is one of these cameras and appears to be the best-suited solution to robotic applications. Like the color filter arrays, this technology seems to have reached significant maturity in terms of performance and repeatability of the measurement, such that its use could be generalized in the future. A variation of this technology also makes it possible to make a joint acquisition of color and polarization images. In our opinion, an effort toward standardization and the definition of dedicated preprocessing pipelines remains to be made, possibly with open-source software toolkits.

**Figure 19:**
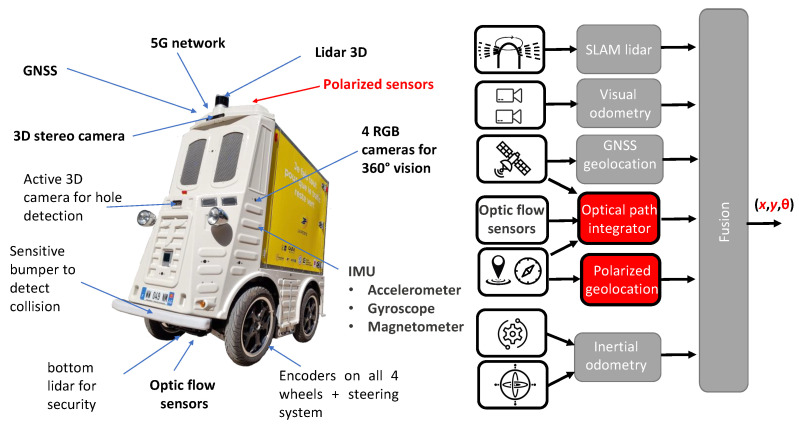
Logistics droid ciTHy L from TwinswHeel (payload up to 300 kg). This delivery droid is currently equipped with an Integrated Navigation System (INS) based on a triple redundancy of locations: 1st 3D Lidar, 2nd Stereo Camera, and 3rd GNSS + IMU + 4 wheels with encoders. The optical path integrator + polarized geolocation will be the 4th redundancy of location to make the robot geolocation more robust in all weather conditions and complex environments. The ciTHy L picture is courtesy of Vincent and Benjamin Talon, Co-founders of TwinswHeel (https://www.twinswheel.fr/, accessed on 19 March 2024).

There are several advantages to using sky polarization for robotics navigation: this technology is undetectable, it has immunity to GNSS signal spoofing or jamming, the celestial heading detection estimate is driftless, and it could work at night by moonlight, making it exploitable in urban environments for civilian applications such as automated last-mile delivery service. An autonomous vehicle such as that proposed by the French company TwinswHeel (Figure 19) could use polarization for guidance as early as 2030.

The polarimetric systems described in this manuscript can estimate geolocation with a sufficient precision using only skylight, and these systems are so lightweight and inexpensive that they could be embedded into terrestrial, aerial, or underwater autonomous vehicles. Improvements of the technology will enable such vehicles to operate using, for instance, the detection of the multispectral polarization patterns. But UV usage, detection of surrounding light in panoramic view and operation in complex weather conditions remain challenges.

Moreover, current popular polarimetric sensors are megapixel cameras, which are too bulky and expensive for applications in automotive or service robotics. An alternative could take its inspiration from nature: some animals detect the celestial heading with a visual system corresponding to the equivalent of very low-definition sensors, which was corroborated by simulations with neural networks using low-definition images. Therefore, an artificial retina, consisting of one thousand pixels (instead of one million pixels for a classical camera) and a dedicated trained processing unit could be the first step toward a low-cost polarimetric device aimed at autonomous navigation.

In this review, we also presented the benefits of polarimetric imaging for robots to help them better understand the world in which they will operate. The detection of transparent or potentially dangerous surfaces can be facilitated by analyzing the polarization of light reflected from surfaces. In an even more advanced way, we have seen how the 3D shape of objects can be estimated from the measurement of polarization parameters. Algorithms based on neural networks can now overcome the constraints associated with shape-from-polarization techniques, making it possible to generalize the reconstruction of objects outdoors under a variety of lighting conditions.

Autonomous robots working in urban environments, e.g., for last-mile delivery services, will have to locate and position themselves with a spatial accuracy of better than 5 cm and 0.2 degree by 2030. Concurrently, in public areas, they must meet the most stringent safety requirements. Using and fusing the polarized sensors’ outputs with an INS could be a supplemental perceptive modality of localization techniques to reach the requested level of performances in order to complement and reinforce conventional localization techniques (3D LiDAR-based SLAM, GNSS, and visual–inertial odometry, see Figure 19).

## Figures and Tables

**Table 1:** Pros and cons of various imaging Stokes polarimeter architectures (inspired from [47]). For two configurations (RoAp and DoAp, labeled with an ${‘}^{*}$’), obtaining only 3 Stokes parameters is reported, but obtaining 4 seems reasonably straightforward. For the Division of Focal plane, most systems provide 3 Stokes parameters; getting the fourth parameter is not straightforward but has been demonstrated in prototype polarimeters.

Type	Technology	Cameras/ Optics	Pros	Cons	Stokes Components
Division of Time (DoT)	Rotating elements [48,49,50]	1/1	Robust Efficient Fairly compact Moderate cost	Requires several acquisitions Cannot process dynamic scenes May require software registration of images	3–4
	Liquid crystal cells [51,52,53,54,55,56,57,58,59]	1/1	Can process dynamic scenes Efficient Fairly compact	Require several acquisitions Dynamic scenes may result in polarimetric artifacts Liquid crystal must be finely characterized and controlled	2–4
Replication of Aperture (RoAp)	Multiple systems [60,61,62]	mult./mult.	Snapshot acquisition	Expensive Images must be registered (mechanically or via software)	3 ($4^{*}$)
Division of Amplitude (DoAmp)	Several focal plane arrays [63,64,65]	multiple/1	Snapshot acquisition Optimum use of light whith polarizing beamsplitters	Bulky system Expensive FPAs must be registered (mechanically or via software)	4
Division of Aperture (DoAp)	One focal plane array [66]	1/1	Snapshot acquisition Compact setup	Loss of spatial resolution Images must be registered (mechanically or via software)	3 ($4^{*}$)
Division of Focal Plane (DoFP)	Polarimetric filter array [67,68,69,70]	1/1	Snapshot acquisition Compact setup Off the shelf devices Low cost	Loss of spatial resolution Requires spatial interpolation to reduce Instantaneous Field of View (IFoV) errors	3

**Table 2:** Evolution of Shape from Polarization in the literature.

	Material		Reflection		Lighting Invariance	Ambiguity Solving		Unknown Refractive Index
	Dielectric	Metallic	Specular	Diffuse		Azimuth	Zenith	
Rahmann 2001 [304]	✓		✓			quadric object, 2 views	-	✓
Miyazaki 2002 [308]	✓		✓			convex shape	visible and IR	
Miyazaki 2004 [278]	✓		✓			convex shape	2 views	
Atkinson 2006 [306]	✓			✓		convex shape	no ambiguity in diffuse mode	
Morel 2006 [279]		✓				active lighting	smooth surfaces	
Huynh 2010 [309]	✓			✓		spectral variation of the phase of polarization	convex surface	✓
Mahmoud 2012 [311]	✓			✓		shape from shading		
Ngo 2015 [312]	✓			✓		shape from shading and controlled lighting		✓
Smith 2016 [313]	✓		✓	✓		shape from shading		
Smith 2018 [316]	✓		✓	✓	✓	shape from shading		
Ba 2020 [317]	✓		✓	✓	✓	shape from shading and deep learning		
Yang 2023 [318]	✓		✓	✓	✓	deep learning		✓
Cai 2023 [320]	✓			✓		Prior feature information of facial polarization images		

## Data Availability

Not applicable.

## Abbreviations

The following abbreviations are used in this manuscript:

AoLP	Angle of Linear Polarization (sometimes referred to as *Angle of Polarization*)
APMR	Accuracy of Polarization Measurement Redundancy
BM3D	Block-Matching and 3D filtering
DoA	Division-of-Aperture
DoFP	Division-of-Focal Plane
DoLP	Degree of Linear Polarization
DoP	Degree of Polarization
DoT	Division-Of-Time
DRA	Dorsal Rim Area
ENU	East North Up
fps	frames per second
GNSS	Global Navigation Satellite System
GPS	Global Positioning System
IFoV	Instantaneous Field of View
INS	Inertial Navigation System
ISO	International Organization for Standardization
NCP	North Celestial Pole
PbC	Polarization-based Compass
PFA	Polarimetric Filter Array
PG	Polarization Gratings
PSA	Polarization State Analyzer
RGB	Red Green Blue
RMSE	Root Mean Squared Error
SLAM	Simultaneous Localization And Mapping
SNR	Signal-to-Noise Ratio
UAV	Unmanned Aerial Vehicle
UV	UltraViolet

## References

[1] Yang G.Z., Bellingham J., Dupont P.E., Fischer P., Floridi L., Full R., Jacobstein N., Kumar V., McNutt M., Merrifield R. (2018). The grand challenges of science robotics. Sci. Robot..

[2] Horváth G., Lerner A., Shashar N. (2014). Polarized Light and Polarization Vision in Animal Sciences.

[3] Able K., Able M. (1995). Manipulations of polarized skylight calibrate magnetic orientation in a migratory bird. J. Comp. Phys. A.

[4] Cochran W.W., Mouritsen H., Wikelski M. (2004). Migrating Songbirds Recalibrate Their Magnetic Compass Daily from Twilight Cues. Science.

[5] Akesson S. (2014). The Ecology of Polarisation Vision in Birds. Polarized Light and Polarization Vision in Animal Sciences.

[6] Wehner R. (2020). Desert Navigator: The Journey of an Ant.

[7] Pieron H. (1904). Du rôle du sens musculaire dans l’orientation de quelques espèces de fourmis. Bull. Inst. Gen. Psychol..

[8] Santschi F. (1911). Observations et remarques critiques sur le mécanisme de l’orientation chez les fourmis. Rev. Suisse Zool..

[9] Papi F. (2001). Animal navigation at the end of the century: A retrospect and a look forward. Ital. J. Zool..

[10] Lambrinos D., Möller R., Labhart T., Pfeifer R., Wehner R. (2000). A mobile robot employing insect strategies for navigation. Robot. Auton. Syst..

[11] Dupeyroux J., Serres J.R., Viollet S. (2019). AntBot: A six-legged walking robot able to home like desert ants in outdoor environments. Sci. Robot..

[12] Dupeyroux J., Viollet S., Serres J.R. (2019). An ant-inspired celestial compass applied to autonomous outdoor robot navigation. Robot. Auton. Syst..

[13] Barta A., Suhai B., Horváth G. (2014). Polarization Cloud Detection with Imaging Polarimetry. Polarized Light and Polarization Vision in Animal Sciences.

[14] Hegedüs R., Åkesson S., Wehner R., Horváth G. (2007). Could Vikings have navigated under foggy and cloudy conditions by skylight polarization? On the atmospheric optical prerequisites of polarimetric Viking navigation under foggy and cloudy skies. Proc. R. Soc. A Math. Phys. Eng. Sci..

[15] Horváth G., Barta A., Pomozi I., Suhai B., Hegedüs R., Åkesson S., Meyer-Rochow B., Wehner R. (2011). On the trail of Vikings with polarized skylight: Experimental study of the atmospheric optical prerequisites allowing polarimetric navigation by Viking seafarers. Philos. Trans. R. Soc. B Biol. Sci..

[16] Ropars G., Gorre G., Le Floch A., Enoch J., Lakshminarayanan V. (2012). A depolarizer as a possible precise sunstone for Viking navigation by polarized skylight. Proc. R. Soc. A Math. Phys. Eng. Sci..

[17] Takacs P., Szaz D., Pereszlenyi A., Horvath G. (2023). Speedy bearings to slacked steering: Mapping the navigation patterns and motions of Viking voyages. PLoS ONE.

[18] (2023). Industrial Trucks—Safety Requirements and Verification—Part 4: Driverless Industrial Trucks and Their Systems.

[19] (2018). Road Vehicles—Functional Safety—Part 1: Vocabulary.

[20] (2022). Road Vehicles—Safety of the Intended Functionality.

[21] Li S., Kong F., Xu H., Guo X., Li H., Ruan Y., Cao S., Guo Y. (2023). Biomimetic Polarized Light Navigation Sensor: A Review. Sensors.

[22] Kong F., Guo Y., Zhang J., Fan X., Guo X. (2023). Review on bio-inspired polarized skylight navigation. Chin. J. Aeronaut..

[23] Li Q., Dong L., Hu Y., Hao Q., Wang W., Cao J., Cheng Y. (2023). Polarimetry for bionic geolocation and navigation applications: A review. Remote Sens..

[24] Liu Y., Wenzhou Z., Fan C., Zhang L. A Review of Bionic Polarized Light Localization Methods. Proceedings of the 2023 5th International Conference on Intelligent Control, Measurement and Signal Processing (ICMSP).

[25] Tominaga S., Kimachi A. (2008). Polarization imaging for material classification. Opt. Eng..

[26] Li X., Yan L., Qi P., Zhang L., Goudail F., Liu T., Zhai J., Hu H. (2023). Polarimetric Imaging via Deep Learning: A Review. Remote Sens..

[27] Stokes G.G. (1852). On the composition and resolution of streams of polarized light from different sources. Trans. Camb. Philos. Soc..

[28] Goldstein D.H. (2010). Polarized Light.

[29] Poincaré H. (1892). Théorie Mathématique de la Lumière.

[30] Geek3 Poincaré Sphere. https://commons.wikimedia.org/wiki/File:Poincare-sphere_arrows.svg.

[31] Perrin F. (1942). Polarization of Light Scattered by Isotropic Opalescent Media. J. Chem. Phys..

[32] Mueller H. (1948). The foundation of optics. J. Opt. Soc. Am..

[33] Jones D., Goldstein D., Spaulding J. (2006). Reflective and polarimetric characteristics of urban materials. Polarization: Measurement, Analysis, and Remote Sensing VII; Proceedings of Defense and Security Symposium, Orlando, FL, USA.

[34] Hoover B.G., Tyo J.S. (2007). Polarization components analysis for invariant discrimination. Appl. Opt..

[35] Wang P., Chen Q., Gu G., Qian W., Ren K. (2016). Polarimetric Image Discrimination With Depolarization Mueller Matrix. IEEE Photonics J..

[36] Quéau Y., Leporcq F., Lechervy A., Alfalou A. (2019). Learning to classify materials using Mueller imaging polarimetry. Proceedings of the Fourteenth International Conference on Quality Control by Artificial Vision.

[37] Kupinski M., Li L. (2020). Evaluating the Utility of Mueller Matrix Imaging for Diffuse Material Classification. J. Imaging Sci. Technol..

[38] Pierangelo A., Nazac A., Benali A., Validire P., Cohen H., Novikova T., Ibrahim B.H., Manhas S., Fallet C., Antonelli M.R. (2013). Polarimetric imaging of uterine cervix: A case study. Opt. Express.

[39] Van Eeckhout A., Lizana A., Garcia-Caurel E., Gil J.J., Sansa A., Rodríguez C., Estévez I., González E., Escalera J.C., Moreno I. (2018). Polarimetric imaging of biological tissues based on the indices of polarimetric purity. J. Biophotonics.

[40] Slonaker R., Takano Y., Liou K.N., Ou S.C. (2005). Circular polarization signal for aerosols and clouds. Atmospheric and Environmental Remote Sensing Data Processing and Utilization: Numerical Atmospheric Prediction and Environmental Monitoring; Proceedings of Optics and Photonics 2005, San Diego, CA, USA.

[41] Gassó S., Knobelspiesse K.D. (2022). Circular polarization in atmospheric aerosols. Atmos. Chem. Phys..

[42] Tyo J.S. (1998). Optimum linear combination strategy for an N-channel polarization-sensitive imaging or vision system. JOSA A.

[43] Tyo J.S. (2002). Design of Optimal Polarimeters: Maximization of Signal-to-Noise Ratio and Minimization of Systematic Error. Appl. Opt..

[44] Perkins R., Gruev V. (2010). Signal-to-noise analysis of Stokes parameters in division of focal plane polarimeters. Opt. Express.

[45] Bass M. (2010). Handbook of Optics: Volume ii-Design, Fabrication, and Testing; Sources and Detectors; Radiometry and Photometry.

[46] Mu T., Pacheco S., Chen Z., Zhang C., Liang R. (2017). Snapshot linear-Stokes imaging spectropolarimeter using division-of-focal-plane polarimetry and integral field spectroscopy. Sci. Rep..

[47] Tyo J.S., Goldstein D.L., Chenault D.B., Shaw J.A. (2006). Review of passive imaging polarimetry for remote sensing applications. Appl. Opt..

[48] Voss K.J., Liu Y. (1997). Polarized radiance distribution measurements of skylight. I. System description and characterization. Appl. Opt..

[49] Kreuter A., Zangerl M., Schwarzmann M., Blumthaler M. (2009). All-sky imaging: A simple, versatile system for atmospheric research. Appl. Opt..

[50] Wang Y., Hu X., Lian J., Zhang L., Xian Z., Ma T. (2014). Design of a Device for Sky Light Polarization Measurements. Sensors.

[51] Wolff L.B., Mancini T.A., Pouliquen P., Andreou A.G. (1997). Liquid crystal polarization camera. IEEE Trans. Robot. Autom..

[52] Chipman R.A. (1995). Polarimetry. Handbook of Optics.

[53] Gandorfer A.M. (1999). Ferroelectric retarders as an alternative to piezoelastic modulators for use in solar Stokes vector polarimetry. Opt. Eng..

[54] Blakeney S.L., Day S.E., Stewart J.N. (2002). Determination of unknown input polarisation using a twisted nematic liquid crystal display with fixed components. Opt. Commun..

[55] Pust N.J., Shaw J.A. (2006). Dual-field imaging polarimeter using liquid crystal variable retarders. Appl. Opt..

[56] Gendre L., Foulonneau A., Bigué L. (2010). Imaging linear polarimetry using a single ferroelectric liquid crystal modulator. Appl. Opt..

[57] Lefaudeux N., Lechocinski N., Breugnot S., Clemenceau P. (2008). Compact and robust linear Stokes polarization camera. Polarization: Measurement, Analysis, and Remote Sensing VIII, Proceedings of the SPIE Defense and Security Symposium, Orlando, FL, USA.

[58] Vedel M., Breugnot S., Lechocinski N., Shaw J.A., Tyo J.S. (2011). Full Stokes polarization imaging camera. Proceedings of the Polarization Science and Remote Sensing V.

[59] Zhang Y., Zhao H., Song P., Shi S., Xu W., Liang X. (2014). Ground-based full-sky imaging polarimeter based on liquid crystal variable retarders. Opt. Express.

[60] Horváth G., Barta A., Gál J., Suhai B., Haiman O. (2002). Ground-based full-sky imaging polarimetry of rapidly changing skies and its use for polarimetric cloud detection. Appl. Opt..

[61] Wang D., Liang H., Zhu H., Zhang S. (2014). A Bionic Camera-Based Polarization Navigation Sensor. Sensors.

[62] Fan C., Hu X., Lian J., Zhang L., He X. (2016). Design and Calibration of a Novel Camera-Based Bio-Inspired Polarization Navigation Sensor. IEEE Sens. J..

[63] de Leon E., Brandt R., Phenis A., Virgen M., Shaw J.A., Tyo J.S. (2007). Initial results of a simultaneous Stokes imaging polarimeter. Polarization Science and Remote Sensing III, Proceedings of SPIE Optical Engineering + Applications, San Diego, CA, USA, 29–30 August 2007.

[64] Fujita K., Itoh Y., Mukai T. (2009). Development of simultaneous imaging polarimeter for asteroids. Adv. Space Res..

[65] Gu D.F., Winker B., Wen B., Mansell J., Zachery K., Taber D., Chang T., Choi S., Ma J., Wang X. (2008). Liquid crystal tunable polarization filters for polarization imaging. Liquid Crystals XII.

[66] Pezzaniti J.L., Chenault D.B. (2005). A division of aperture MWIR imaging polarimeter. Polarization Science and Remote Sensing II; Proceedings of Optics and Photonics 2005, San Diego, CA, USA.

[67] Chun C., Fleming D., Torok E. (1994). Polarization-sensitive thermal imaging. Automatic Object Recognition IV; Proceedings of the SPIE’s International Symposium on Optical Engineering and Photonics in Aerospace Sensing, Orlando, FL, USA.

[68] Gruev V., Perkins R., York T. (2010). CCD polarization imaging sensor with aluminum nanowire optical filters. Opt. Express.

[69] Brock N., Kimbrough B., Millerd J. (2011). A pixelated micropolarizer-based camera for instantaneous interferometric measurements. Polarization Science and Remote Sensing V, Proceedings of the SPIE Optical Engineering + Applications Symposium, San Diego, CA, USA.

[70] Sony Semiconductor Solutions Group Polarization Image Sensor Polarsens. https://www.sony-semicon.com/files/62/flyer_industry/IMX250_264_253MZR_MYR_Flyer_en.pdf.

[71] Efron U. (1995). Spatial Light Modulator Technology: Materials, Devices, and Applications.

[72] Jaulin A., Bigué L., Ambs P. (2008). High-speed degree-of-polarization imaging with a ferroelectric liquid-crystal modulator. Opt. Eng..

[73] Gendre L., Foulonneau A., Bigué L. (2011). Full Stokes polarimetric imaging using a single ferroelectric liquid crystal device. Opt. Eng..

[74] Xu C., Ma J., Ke C., Huang Y., Zeng Z., Weng W., Shen L., Wang K. (2020). Full-Stokes polarization imaging based on liquid crystal variable retarders and metallic nanograting arrays. J. Phys. D Appl. Phys..

[75] Harchanko J., Pezzaniti L., Chenault D., Eades G. (2008). Comparing a MWIR and LWIR polarimetric imaging for surface swimmer detection. Optics and Photonics in Global Homeland Security IV, Proceedings of the SPIE Defense and Security Symposium, Orlando, FL, USA.

[76] Shibata S., Suzuki M., Hagen N., Otani Y. (2019). Video-rate full-Stokes imaging polarimeter using two polarization cameras. Opt. Eng..

[77] Gori F. (1999). Measuring Stokes parameters by means of a polarization grating. Opt. Lett..

[78] Rubin N.A., D’Aversa G., Chevalier P., Shi Z., Chen W.T., Capasso F. (2019). Matrix Fourier optics enables a compact full-Stokes polarization camera. Science.

[79] Kim J., Escuti M.J. (2008). Snapshot imaging spectropolarimeter utilizing polarization gratings. Proceedings of the Imaging Spectrometry XIII, Proceedings of Optical Engineering + Applications, San Diego, CA, USA.

[80] Bayer B.E. (1976). Color Imaging Array. United States Patent.

[81] Daly I., How M., Partridge J., Temple S., Marshall N., Cronin T., Roberts N. (2016). Dynamic polarization vision in mantis shrimps. Nat. Commun..

[82] How M. Polarization Anatomy of a Mantis Shrimp Eye. https://commons.wikimedia.org/wiki/File:Polarization_anatomy_of_a_mantis_shrimp_eye.png.

[83] Gimenez Y. (2022). Characterization of Stokes Imaging Systems Using Micropolarizers Filters Arrays. Ph.D. Thesis.

[84] Powell S.B., Gruev V. (2013). Calibration methods for division-of-focal-plane polarimeters. Opt. Express.

[85] Hagen N., Shibata S., Otani Y. (2019). Calibration and performance assessment of microgrid polarization cameras. Opt. Eng..

[86] Fei H., Li F.M., Chen W.C., Zhang R., Chen C.S. (2018). Calibration method for division of focal plane polarimeters. Appl. Opt..

[87] Gimenez Y., Lapray P.J., Foulonneau A., Bigué L. (2020). Calibration algorithms for polarization filter array camera: Survey and evaluation. J. Electron. Imaging.

[88] Wu R., Zhao Y., Li N., Kong S.G. (2021). Polarization image demosaicking using polarization channel difference prior. Opt. Express.

[89] Lane C., Rode D., Rösgen T. (2022). Calibration of a polarization image sensor and investigation of influencing factors. Appl. Opt..

[90] Mihoubi S., Lapray P.J., Bigué L. (2018). Survey of Demosaicking Methods for Polarization Filter Array Images. Sensors.

[91] Li N., Zhao Y., Pan Q., Kong S.G. (2019). Demosaicking DoFP images using Newton’s polynomial interpolation and polarization difference model. Opt. Express.

[92] Morimatsu M., Monno Y., Tanaka M., Okutomi M. (2021). Monochrome and Color Polarization Demosaicking Based on Intensity-Guided Residual Interpolation. IEEE Sens. J..

[93] Pistellato M., Bergamasco F., Fatima T., Torsello A. (2022). Deep Demosaicing for Polarimetric Filter Array Cameras. IEEE Trans. Image Process..

[94] Li N., Zhao Y., Pan Q., Kong S.G., Chan J.C.W. (2020). Full-time monocular road detection using zero-distribution prior of angle of polarization. Proceedings of the Computer Vision–ECCV 2020: 16th European Conference.

[95] Blin R., Ainouz S., Canu S., Meriaudeau F. Multimodal Polarimetric And Color Fusion For Road Scene Analysis In Adverse Weather Conditions. Proceedings of the 2021 IEEE International Conference on Image Processing (ICIP).

[96] Courtier G., Adam R., Lapray P.J., Pecheur E., Changey S., Lauffenburger J.P. (2022). Image-based navigation system using skylight polarization for an unmanned ground vehicle. Unmanned Systems Technology XXIV; SPIE Defense + Commercial Sensing, Orlando, FL, USA.

[97] Onuma T., Otani Y. (2014). A development of two-dimensional birefringence distribution measurement system with a sampling rate of 1.3MHz. Opt. Commun..

[98] Wu X., Pankow M., Onuma T., Huang H.Y.S., Peters K. (2022). Comparison of High-Speed Polarization Imaging Methods for Biological Tissues. Sensors.

[99] Qi J., He C., Elson D.S. (2017). Real time complete Stokes polarimetric imager based on a linear polarizer array camera for tissue polarimetric imaging. Biomed. Opt. Express.

[100] Myhre G., Hsu W.L., Peinado A., LaCasse C., Brock N., Chipman R.A., Pau S. (2012). Liquid crystal polymer full-stokes division of focal plane polarimeter. Opt. Express.

[101] LeMaster D.A., Hirakawa K. (2014). Improved microgrid arrangement for integrated imaging polarimeters. Opt. Lett..

[102] Alenin A.S., Vaughn I.J., Tyo J.S. (2017). Optimal bandwidth micropolarizer arrays. Opt. Lett..

[103] Alenin A.S., Vaughn I.J., Tyo J.S. (2018). Optimal bandwidth and systematic error of full-Stokes micropolarizer arrays. Appl. Opt..

[104] Hoover B.G., Rugely D.A., Francis C.M., Zeira G., Gamiz V.L. (2016). Bistatic laser polarimeter calibrated to 1% at visible-SWIR wavelengths. Opt. Express.

[105] Boulbry B., Ramella-Roman J.C., Germer T.A. (2007). Improved method for calibrating a Stokes polarimeter. Appl. Opt..

[106] Compain E., Poirier S., Drevillon B. (1999). General and self-consistent method for the calibration of polarization modulators, polarimeters, and Mueller-matrix ellipsometers. Appl. Opt..

[107] Mu T., Bao D., Zhang C., Chen Z., Song J. (2018). Optimal reference polarization states for the calibration of general Stokes polarimeters in the presence of noise. Opt. Commun..

[108] Goudail F. (2009). Noise minimization and equalization for Stokes polarimeters in the presence of signal-dependent Poisson shot noise. Opt. Lett..

[109] Roussel S., Boffety M., Goudail F. (2018). Polarimetric precision of micropolarizer grid-based camera in the presence of additive and Poisson shot noise. Opt. Express.

[110] Giménez Y., Lapray P.J., Foulonneau A., Bigué L. (2019). Calibration for polarization filter array cameras: Recent advances. Proceedings of the Fourteenth International Conference on Quality Control by Artificial Vision, Mulhouse, France.

[111] Morel O., Seulin R., Fofi D. (2016). Handy method to calibrate division-of-amplitude polarimeters for the first three Stokes parameters. Opt. Express.

[112] Rodriguez J., Lew-Yan-Voon L., Martins R., Morel O. (2022). A Practical Calibration Method for RGB Micro-Grid Polarimetric Cameras. IEEE Robot. Autom. Lett..

[113] Le Teurnier B., Li N., Boffety M., Goudail F. (2022). Definition of an error map for DoFP polarimetric images and its application to retardance calibration. Opt. Express.

[114] Tyo J.S., LaCasse C.F., Ratliff B.M. (2009). Total elimination of sampling errors in polarization imagery obtained with integrated microgrid polarimeters. Opt. Lett..

[115] Ratliff B.M., LaCasse C.F., Scott Tyo J. (2009). Interpolation strategies for reducing IFOV artifacts in microgrid polarimeter imagery. Opt. Express.

[116] Le Teurnier B., Boffety M., Goudail F. (2022). Error model for linear DoFP imaging systems perturbed by spatially varying polarization states. Appl. Opt..

[117] Gao S., Gruev V. (2011). Bilinear and bicubic interpolation methods for division of focal plane polarimeters. Opt. Express.

[118] Ratliff B.M., LaCasse C.F., Tyo J.S. Adaptive strategy for demosaicing microgrid polarimeter imagery. Proceedings of the 2011 Aerospace Conference.

[119] Zhang J., Luo H., Hui B., Chang Z. (2016). Image interpolation for division of focal plane polarimeters with intensity correlation. Opt. Express.

[120] Morimatsu M., Monno Y., Tanaka M., Okutomi M. Monochrome And Color Polarization Demosaicking Using Edge-Aware Residual Interpolation. Proceedings of the 2020 IEEE International Conference on Image Processing (ICIP).

[121] Ratliff B.M., Tyo J.S., Black W.T., LaCasse C.F., Shaw J.A., Tyo J.S. (2009). Exploiting motion-based redundancy to enhance microgrid polarimeter imagery. Polarization Science and Remote Sensing IV, Proceedings of SPIE Optical Engineering + Applications Symposium.

[122] Hardie R.C., LeMaster D.A., Ratliff B.M. (2011). Super-resolution for imagery from integrated microgrid polarimeters. Opt. Express.

[123] Zhang J., Luo H., Liang R., Ahmed A., Zhang X., Hui B., Chang Z. (2018). Sparse representation-based demosaicing method for microgrid polarimeter imagery. Opt. Lett..

[124] Wen S., Zheng Y., Lu F., Zhao Q. (2019). Convolutional demosaicing network for joint chromatic and polarimetric imagery. Opt. Lett..

[125] Nguyen V., Tanaka M., Monno Y., Okutomi M. Two-Step Color-Polarization Demosaicking Network. Proceedings of the 2022 IEEE International Conference on Image Processing (ICIP).

[126] Smith M., Woodruff J., Howe J. (1999). Beam wander considerations in imaging polarimetry. Polarization: Measurement, Analysis, and Remote Sensing II, Proceedings of the SPIE’s International Symposium on Optical Science, Engineering, and Instrumentation, Denver, CO, USA.

[127] Guizar-Sicairos M., Thurman S.T., Fienup J.R. (2008). Efficient subpixel image registration algorithms. Opt. Lett..

[128] Bigué L., Foulonneau A., Lapray P.J. (2023). Production of high-resolution reference polarization images from real world scenes. Polarization Science and Remote Sensing XI, Proceedings of SPIE Optics + Photonics symposium, San Diego, CA, USA, 21–22 August 2023.

[129] Zeng X., Luo Y., Zhao X., Ye W. (2019). An end-to-end fully-convolutional neural network for division of focal plane sensors to reconstruct S0, DoLP, and AoP. Opt. Express.

[130] Guyot S., Anastasiadou M., Deléchelle E., De Martino A. (2007). Registration scheme suitable to Mueller matrix imaging for biomedical applications. Opt. Express.

[131] Marconnet P., Gendre L., Foulonneau A., Bigué L., Shaw J.A., Tyo J.S. (2011). Cancellation of motion artifacts caused by a division-of-time polarimeter. Polarization Science and Remote Sensing V, Proceedings of SPIE Optical Engineering + Applications Symposium, San Diego, CA, USA, 21–22 August 2011.

[132] Goldstein D.H., Chipman R.A. (1990). Error analysis of a Mueller matrix polarimeter. J. Opt. Soc. Am. A.

[133] Sabatke D.S., Descour M.R., Dereniak E.L., Sweatt W.C., Kemme S.A., Phipps G.S. (2000). Optimization of retardance for a complete Stokes polarimeter. Opt. Lett..

[134] Goudail F., Bénière A. (2010). Estimation precision of the degree of linear polarization and of the angle of polarization in the presence of different sources of noise. Appl. Opt..

[135] Tibbs A.B., Daly I.M., Bull D.R., Roberts N.W. (2018). Noise creates polarization artefacts. Bioinspir. Biomim..

[136] Li N., Le Teurnier B., Boffety M., Goudail F., Zhao Y., Pan Q. (2021). No-Reference Physics-Based Quality Assessment of Polarization Images and Its Application to Demosaicking. IEEE Trans. Image Process..

[137] Dabov K., Foi A., Katkovnik V., Egiazarian K. (2007). Image Denoising by Sparse 3-D Transform-Domain Collaborative Filtering. IEEE Trans. Image Process..

[138] Tibbs A.B., Daly I.M., Roberts N.W., Bull D.R. (2018). Denoising imaging polarimetry by adapted BM3D method. J. Opt. Soc. Am. A.

[139] Shibata S., Hagen N., Otani Y. (2019). Robust full Stokes imaging polarimeter with dynamic calibration. Opt. Lett..

[140] Zhao Y., Peng Q., Yi C., Kong S.G. (2016). Multiband Polarization Imaging. J. Sens..

[141] Farlow C.A., Chenault D.B., Pezzaniti J.L., Spradley K.D., Gulley M.G. (2002). Imaging polarimeter development and applications. Polarization Analysis and Measurement IV, Proceedings of the International Symposium on Optical Science and Technology, San Diego, CA, USA, 29 July–3 August 2001.

[142] Alouini M., Goudail F., Réfrégier P., Grisard A., Lallier E., Dolfi D., Goldstein D.H., Chenault D.B. (2004). Multispectral polarimetric imaging with coherent illumination: Towards higher image contrast. Polarization: Measurement, Analysis, and Remote Sensing VI, Proceedings of the Defense and Security Symposium, Orlando, FL, USA, 15 April 2004.

[143] Twede D. (2013). Single Camera Color and Infrared Polarimetric Imaging. US Patent.

[144] Spote A., Lapray P.J., Thomas J.B., Farup I. (2021). Joint demosaicing of colour and polarisation from filter arrays. Proceedings of the Color and Imaging Conference.

[145] Liu J., Duan J., Hao Y., Chen G., Zhang H., Zheng Y. (2023). Polarization image demosaicing and RGB image enhancement for a color polarization sparse focal plane array. Opt. Express.

[146] Tu X., Spires O.J., Tian X., Brock N., Liang R., Pau S. (2017). Division of amplitude RGB full-Stokes camera using micro-polarizer arrays. Opt. Express.

[147] Kurita T., Kondo Y., Sun L., Moriuchi Y. Simultaneous Acquisition of High Quality RGB Image and Polarization Information using a Sparse Polarization Sensor. Proceedings of the 2023 IEEE/CVF Winter Conference on Applications of Computer Vision (WACV).

[148] Zou X., Gong G., Lin Y., Fu B., Wang S., Zhu S., Wang Z. (2023). Metasurface-based polarization color routers. Opt. Lasers Eng..

[149] Garcia M., Edmiston C., Marinov R., Vail A., Gruev V. (2017). Bio-inspired color-polarization imager for real-time in situ imaging. Optica.

[150] Garcia M., Davis T., Blair S., Cui N., Gruev V. (2018). Bioinspired polarization imager with high dynamic range. Optica.

[151] Altaqui A., Sen P., Schrickx H., Rech J., Lee J.W., Escuti M., You W., Kim B.J., Kolbas R., O’Connor B.T. (2021). Mantis shrimp–inspired organic photodetector for simultaneous hyperspectral and polarimetric imaging. Sci. Adv..

[152] Han F., Mu T., Li H., Tuniyazi A. (2023). Deep image prior plus sparsity prior: Toward single-shot full-Stokes spectropolarimetric imaging with a multiple-order retarder. Adv. Photonics Nexus.

[153] Schechner Y.Y., Narasimhan S.G., Nayar S.K. (2003). Polarization-based vision through haze. Appl. Opt..

[154] Geng Y., Kizhakidathazhath R., Lagerwall J.P.F. (2021). Encoding Hidden Information onto Surfaces Using Polymerized Cholesteric Spherical Reflectors. Adv. Funct. Mater..

[155] Tu X., McEldowney S., Zou Y., Smith M., Guido C., Brock N., Miller S., Jiang L., Pau S. (2020). Division of focal plane red–green–blue full-Stokes imaging polarimeter. Appl. Opt..

[156] Maeda R. Polanalyser. https://github.com/elerac/polanalyser.

[157] Rodriguez J., Lew-Yan-Voon L.F.C., Martins R., Morel O. (2024). Pola4All: Survey of polarimetric applications and an open-source toolkit to analyze polarization. J. Electron. Imaging.

[158] Moody L.C.A.B. (1950). The pfund sky compass. Navig. J. Inst. Navig..

[159] Aycock T., Lompado A., Wolz T., Chenault D. (2016). Passive optical sensing of atmospheric polarization for GPS denied operations. Proceedings of the Sensors and Systems for Space Applications IX.

[160] Aycock T.M., Chenault D., Lompado A., Pezzaniti J.L. (2016). Sky Polarization and Sun Sensor System and Method. US Patent.

[161] Aycock T.M., Chenault D.B., Lompado A., Pezzaniti J.L. (2018). Sky Polarization and Sun Sensor System and Method. US Patent.

[162] Eshelman L.M., Smith A.M., Smith K.M., Chenault D.B. (2022). Unique navigation solution utilizing sky polarization signatures. Proceedings of the Polarization: Measurement, Analysis, and Remote Sensing XV.

[163] Hamaoui M. (2017). Polarized skylight navigation. Appl. Opt..

[164] Dupeyroux J., Viollet S., Serres J.R. (2020). Bio-inspired celestial compass yields new opportunities for urban localization. Proceedings of the 2020 28th Mediterranean Conference on Control and Automation (MED).

[165] Courtier G., Lapray P.J., Adam R., Changey S., Lauffenburger J.P. (2023). Ground Vehicle Navigation Based on the Skylight Polarization. Proceedings of the 2023 IEEE/ION Position, Location and Navigation Symposium (PLANS).

[166] Stürzl W., Carey N., Fusiello A., Murino V., Cucchiara R. (2012). A Fisheye Camera System for Polarisation Detection on UAVs. Proceedings of the Computer Vision—ECCV 2012. Workshops and Demonstrations.

[167] Gkanias E., Mitchell R., Stankiewicz J., Khan S.R., Mitra S., Webb B. (2023). Celestial compass sensor mimics the insect eye for navigation under cloudy and occluded skies. Commun. Eng..

[168] Fan C., Hu X., He X., Zhang L., Wang Y. (2018). Multicamera polarized vision for the orientation with the skylight polarization patterns. Opt. Eng..

[169] Fan Y., Zhang R., Liu Z., Chu J. (2021). A skylight orientation sensor based on S-waveplate and linear polarizer for autonomous navigation. IEEE Sens. J..

[170] Yang J., Du T., Niu B., Li C., Qian J., Guo L. (2018). A bionic polarization navigation sensor based on polarizing beam splitter. IEEE Access.

[171] Zhao H., Xu W. (2016). A bionic polarization navigation sensor and its calibration method. Sensors.

[172] Zhao H., Xu W., Zhang Y., Li X., Zhang H., Xuan J., Jia B. (2018). Polarization patterns under different sky conditions and a navigation method based on the symmetry of the AOP map of skylight. Opt. Express.

[173] Guan L., Li S., Zhai L., Liu S., Liu H., Lin W., Cui Y., Chu J., Xie H. (2018). Study on skylight polarization patterns over the ocean for polarized light navigation application. Appl. Opt..

[174] Guan L., Zhai L., Cai H., Zhang P., Li Y., Chu J., Jin R., Xie H. (2020). Study on displacement estimation in low illumination environment through polarized contrast-enhanced optical flow method for polarization navigation applications. Optik.

[175] He R., Hu X., Zhang L., He X., Han G. (2019). A combination orientation compass based on the information of polarized skylight/geomagnetic/MIMU. IEEE Access.

[176] Guo X., Chu J., Wang Y., Wan Z., Li J., Lin M. (2019). Formation experiment with heading angle reference using sky polarization pattern at twilight. Appl. Opt..

[177] Wang Y., Chu J., Zhang R., Li J., Guo X., Lin M. (2019). A bio-inspired polarization sensor with high outdoor accuracy and central-symmetry calibration method with integrating sphere. Sensors.

[178] Liang H., Bai H., Liu N., Sui X. (2020). Polarized skylight compass based on a soft-margin support vector machine working in cloudy conditions. Appl. Opt..

[179] Li J., Chu J., Zhang R., Chen J., Wang Y. (2020). Bio-inspired attitude measurement method using a polarization skylight and a gravitational field. Appl. Opt..

[180] Yang J., Liu X., Zhang Q., Du T., Guo L. (2020). Global autonomous positioning in GNSS-challenged environments: A bioinspired strategy by polarization pattern. IEEE Trans. Ind. Electron..

[181] Yang Y., Hu P., Yang J., Wang S., Zhang Q., Wang Y. (2020). Clear night sky polarization patterns under the super blue blood moon. Atmosphere.

[182] Zhang J., Yang J., Wang S., Liu X., Wang Y., Yu X. (2021). A self-contained interactive iteration positioning and orientation coupled navigation method based on skylight polarization. Control Eng. Pract..

[183] Wan Z., Zhao K., Chu J. (2021). A Novel Attitude Measurement Method Based on Forward Polarimetric Imaging of Skylight. IEEE Trans. Instrum. Meas..

[184] Strutt J. (1871). LVIII. On the scattering of light by small particles. Lond. Edinb. Dublin Philos. Mag. J. Sci..

[185] Strutt J. (1871). XV. On the light from the sky, its polarization and colour. Lond. Edinb. Dublin Philos. Mag. J. Sci..

[186] Coulson K.L. (1959). Characteristics of the radiation emerging from the top of a rayleigh atmosphere—I: Intensity and polarization. Planet. Space Sci..

[187] Coulson K.L. (1988). Polarization and Intensity of Light in the Atmosphere.

[188] Gál J., Horváth G., Barta A., Wehner R. (2001). Polarization of the moonlit clear night sky measured by full-sky imaging polarimetry at full Moon: Comparison of the polarization of moonlit and sunlit skies. J. Geophys. Res. Atmos..

[189] Brines M.L., Gould J.L. (1982). Skylight polarization patterns and animal orientation. J. Exp. Biol..

[190] Eshelman L.M., Shaw J.A. (2019). Visualization of all-sky polarization images referenced in the instrument, scattering, and solar principal planes. Opt. Eng..

[191] Berry M., Dennis M., Lee R. (2004). Polarization singularities in the clear sky. New J. Phys..

[192] Wang X., Gao J., Fan Z., Roberts N.W. (2016). An analytical model for the celestial distribution of polarized light, accounting for polarization singularities, wavelength and atmospheric turbidity. J. Opt..

[193] Moutenet A., Poughon L., Toulon B., Serres J.R., Viollet S. (2024). OpenSky: A modular and open-source simulator of sky polarization measurements. IEEE Trans. Instrum. Meas..

[194] Cornet C., C-Labonnote L., Szczap F. (2010). Three-dimensional polarized Monte Carlo atmospheric radiative transfer model (3DMCPOL): 3D effects on polarized visible reflectances of a cirrus cloud. J. Quant. Spectrosc. Radiat. Transf..

[195] Sheppard P.A. (1961). Tables Related to Radiation Emerging from a Planetary Atmosphere with Rayleigh Scattering K. L. Coulson, J. V. Dave; Z. Sekera (8½ in. × 11 in., xii + 548 pp., University of California Press, 1960). Geophys. J. Int..

[196] Horváth G., Bernáth B., Suhai B., Barta A., Wehner R. (2002). First observation of the fourth neutral polarization point in the atmosphere. JOSA A.

[197] Horváth G., Varjú D. (2004). Polarized Light in Animal Vision: Polarization Patterns in Nature.

[198] Yan L., Yang B., Zhang F., Xiang Y., Chen W. (2020). Atmospheric Remote Sensing 2: Neutral Point Areas of Atmospheric Polarization and Land-Atmosphere Parameter Separation. Polarization Remote Sensing Physics.

[199] Li G., Zhang Y., Fan S., Wang Y., Yu F. (2022). Robust Heading Measurement Based on Improved Berry Model for Bionic Polarization Navigation. IEEE Trans. Instrum. Meas..

[200] Jue W., Pengwei H., Jianqiang Q., Lei G. (2023). Confocal Ellipse Hough Transform for Polarization Compass in the Nonideal Atmosphere. IEEE Trans. Instrum. Meas..

[201] Fan Z., Wang X., Jin H., Wang C., Pan N., Hua D. (2021). Neutral point detection using the AOP of polarized skylight patterns. Opt. Express.

[202] Bellver C. (1987). Influence of particulate pollution on the positions of neutral points in the sky at Seville (Spain). Atmos. Environ. (1967).

[203] Pan P., Wang X., Yang T., Pu X., Wang W., Bao C., Gao J. (2023). High-similarity analytical model of skylight polarization pattern based on position variations of neutral points. Opt. Express.

[204] Labhart T. (1988). Polarization-opponent interneurons in the insect visual system. Nature.

[205] Lambrinos D., Kobayashi H., Pfeifer R., Maris M., Labhart T., Wehner R. (1997). An autonomous agent navigating with a polarized light compass. Adapt. Behav..

[206] Dupeyroux J., Viollet S., Serres J.R. (2019). Polarized skylight-based heading measurements: A bio-inspired approach. J. R. Soc. Interface.

[207] Wang Y., Chu J., Zhang R., Wang L., Wang Z. (2015). A novel autonomous real-time position method based on polarized light and geomagnetic field. Sci. Rep..

[208] Zhi W., Chu J., Li J., Wang Y. (2018). A novel attitude determination system aided by polarization sensor. Sensors.

[209] Yang J., Du T., Liu X., Niu B., Guo L. (2019). Method and implementation of a bioinspired polarization-based attitude and heading reference system by integration of polarization compass and inertial sensors. IEEE Trans. Ind. Electron..

[210] Qiu Z., Wang S., Hu P., Guo L. (2023). Outlier-Robust Extended Kalman Filtering for Bioinspired Integrated Navigation System. IEEE Trans. Autom. Sci. Eng..

[211] Zhao D., Liu Y., Wu X., Dong H., Wang C., Tang J., Shen C., Liu J. (2022). Attitude-Induced error modeling and compensation with GRU networks for the polarization compass during UAV orientation. Measurement.

[212] Liang H., Bai H., Zhou T. (2020). Exploration of Whether Skylight Polarization Patterns Contain Three-dimensional Attitude Information. arXiv.

[213] Pan S., Lin J., Zhang Y., Hu B., Liu X., Yu Q. (2024). Image-registration-based solar meridian detection for accurate and robust polarization navigation. Opt. Express.

[214] Fan C., Hu X., He X., Zhang L., Lian J. (2017). Integrated polarized skylight sensor and MIMU with a metric map for urban ground navigation. IEEE Sens. J..

[215] Collett M., Collett T.S., Bisch S., Wehner R. (1998). Local and global vectors in desert ant navigation. Nature.

[216] Zhou W., Fan C., He X., Hu X., Fan Y., Wu X., Shang H. (2021). Integrated bionic polarized vision/vins for goal-directed navigation and homing in unmanned ground vehicle. IEEE Sens. J..

[217] Han G., Hu X., Lian J., He X., Zhang L., Wang Y., Dong F. (2017). Design and Calibration of a Novel Bio-Inspired Pixelated Polarized Light Compass. Sensors.

[218] Liu X., Yang J., Guo L., Yu X., Wang S. (2021). Design and calibration model of a bioinspired attitude and heading reference system based on compound eye polarization compass. Bioinspir. Biomim..

[219] Ren H., Yang J., Liu X., Huang P., Guo L. (2020). Sensor Modeling and Calibration Method Based on Extinction Ratio Error for Camera-Based Polarization Navigation Sensor. Sensors.

[220] Bai X., Zhu Z., Schwing A., Forsyth D., Gruev V. (2023). Angle of polarization calibration for omnidirectional polarization cameras. Opt. Express.

[221] Urquhart B., Kurtz B., Kleissl J. (2016). Sky camera geometric calibration using solar observations. Atmos. Meas. Tech..

[222] Jin H., Wang X., Fan Z., Pan N. (2021). Linear solution method of solar position for polarized light navigation. IEEE Sens. J..

[223] Poughon L., Aubry V., Monnoyer J., Viollet S., Serres J.R. A stand-alone polarimetric acquisition system for producing a long-term skylight dataset. Proceedings of the 2023 IEEE SENSORS.

[224] Wang Y., Hu X., Lian J., Zhang L., He X. (2017). Bionic orientation and visual enhancement with a novel polarization camera. IEEE Sens. J..

[225] Liu B., Fan Z., Wang X. (2020). Solar position acquisition method for polarized light navigation based on *∞* characteristic model of polarized skylight pattern. IEEE Access.

[226] Guan L., Liu S., Chu J., Zhang R., Chen Y., Li S., Zhai L., Li Y., Xie H. (2019). A novel algorithm for estimating the relative rotation angle of solar azimuth through single-pixel rings from polar coordinate transformation for imaging polarization navigation sensors. Optik.

[227] Zhang W., Zhang X., Cao Y., Liu H., Liu Z. (2016). Robust sky light polarization detection with an S-wave plate in a light field camera. Appl. Opt..

[228] Lyot B. (1944). Le filtre monochromatique polarisant et ses applications en physique solaire. Ann. D’Astrophysique.

[229] Poughon L., Mafrica S., Monnoyer J., Pradere L., Serres J.R., Viollet S. (2021). Procédé et système pour déterminer des données caractérisant un cap Suivi par un véhicule automobile à un instant courant. https://data.inpi.fr/brevets/FR3128528?q=FR3128528#FR3128528.

[230] Poughon L., Aubry V., Monnoyer J., Viollet S., Serres J.R. Skylight polarization heading sensor using waveplate retardance shift with incidence. Proceedings of the Journée des Jeunes Chercheurs en Robotique 2023 (JJCR’23).

[231] Born M., Wolf E. (1999). Principles of Optics.

[232] Labhart T. (1996). How polarization-sensitive interneurones of crickets perform at low degrees of polarization. J. Exp. Biol..

[233] Sakura M., Lambrinos D., Labhart T. (2008). Polarized Skylight Navigation in Insects: Model and Electrophysiology of e-Vector Coding by Neurons in the Central Complex. J. Neurophysiol..

[234] Labhart T. (2016). Can invertebrates see the e-vector of polarization as a separate modality of light?. J. Exp. Biol..

[235] Wang X., Gao J., Fan Z. (2014). Empirical corroboration of an earlier theoretical resolution to the UV paradox of insect polarized skylight orientation. Naturwissenschaften.

[236] Xian Z., Hu X., Lian J., Zhang L., Cao J., Wang Y., Ma T. (2014). A Novel Angle Computation and Calibration Algorithm of Bio-Inspired Sky-Light Polarization Navigation Sensor. Sensors.

[237] Huang X.D., Wang C.H., Pan J.R., Chen J.B., Song C.L., Li L.L. The Error Analysis and the Error Calibration of the Bionic Polarized Light Compass. Proceedings of the 39th Chinese Control Conference (CCC).

[238] Gkanias E., Risse B., Mangan M., Webb B. (2019). From skylight input to behavioural output: A computational model of the insect polarised light compass. PLoS Comput. Biol..

[239] Zhang Q., Yang J., Huang P., Liu X., Wang S., Guo L. (2021). Bionic integrated positioning mechanism based on bioinspired polarization compass and inertial navigation system. Sensors.

[240] Du T., Tian C., Yang J., Wang S., Liu X., Guo L. (2020). An autonomous initial alignment and observability analysis for SINS with bio-inspired polarized skylight sensors. IEEE Sens. J..

[241] Zhang Q., Yang J., Liu X., Guo L. (2020). A bio-inspired navigation strategy fused polarized skylight and starlight for unmanned aerial vehicles. IEEE Access.

[242] Powell S.B., Garnett R., Marshall J., Rizk C., Gruev V. (2018). Bioinspired polarization vision enables underwater geolocalization. Sci. Adv..

[243] Zhao D., Liu X., Zhao H., Wang C., Tang J., Liu J., Shen C. (2021). Seamless integration of polarization compass and inertial navigation data with a self-learning multi-rate residual correction algorithm. Measurement.

[244] Yang J., Wang J., Wang Y., Hu X. (2021). Algorithm design and experimental verification of a heading measurement system based on polarized light/inertial combination. Opt. Commun..

[245] Dou Q., Du T., Qiu Z., Wang S., Yang J. (2022). An adaptive anti-disturbance navigation method for polarized skylight-based autonomous integrated navigation system. Measurement.

[246] Li G., Zhang Y., Fan S., Liu C., Yu F., Wei X., Jin W. (2024). Attitude and heading measurement based on adaptive complementary Kalman filter for PS/MIMU integrated system. Opt. Express.

[247] He X., Zhang L., Fan C., Wang M., Wu W. (2019). A MIMU/Polarized Camera/GNSS Integrated Navigation Algorithm for UAV Application. Proceedings of the 2019 DGON Inertial Sensors and Systems (ISS).

[248] Cao S., Gao H., You J. (2022). In-Flight Alignment of Integrated SINS/GPS/Polarization/Geomagnetic Navigation System Based on Federal UKF. Sensors.

[249] Shen C., Xiong Y., Zhao D., Wang C., Cao H., Song X., Tang J., Liu J. (2022). Multi-rate strong tracking square-root cubature Kalman filter for MEMS-INS/GPS/polarization compass integrated navigation system. Mech. Syst. Signal Process..

[250] Du T., Zeng Y.H., Yang J., Tian C.Z., Bai P.F. (2020). Multi-sensor fusion SLAM approach for the mobile robot with a bio-inspired polarised skylight sensor. IET Radar Sonar Navig..

[251] Du T., Shi S., Zeng Y., Yang J., Guo L. (2022). An integrated INS/LiDAR odometry/polarized camera pose estimation via factor graph optimization for sparse environment. IEEE Trans. Instrum. Meas..

[252] Li J., Chu J., Zhang R., Hu H., Tong K., Li J. (2022). Biomimetic navigation system using a polarization sensor and a binocular camera. JOSA A.

[253] Xia L., Liu R., Zhang D., Zhang J. (2022). Polarized light-aided visual-inertial navigation system: Global heading measurements and graph optimization-based multi-sensor fusion. Meas. Sci. Technol..

[254] Kronland-Martinet T., Poughon L., Pasquinelli M., Duché D., Serres J.R., Viollet S. (2023). SkyPole-A method for locating the north celestial pole from skylight polarization patterns. Proc. Natl. Acad. Sci. USA.

[255] Emlen S. (1972). The Ontogenetic Development of Orientation Capabilities. NASA Spec. Publ..

[256] Brines M. (1980). Dynamic patterns of skylight polarization as clock and compass. J. Theor. Biol..

[257] Waterman T.H. (1954). Polarization patterns in submarine illumination. Science.

[258] Waterman T.H. (2006). Reviving a neglected celestial underwater polarization compass for aquatic animals. Biol. Rev..

[259] Hu P., Yang J., Guo L., Yu X., Li W. (2022). Solar-tracking methodology based on refraction-polarization in Snell’s window for underwater navigation. Chin. J. Aeronaut..

[260] Cheng H., Zhang Q., Wan Z., Zhang Z., Qin J. (2023). Study on the polarization pattern induced by wavy water surfaces. Remote Sens..

[261] Lerner A., Sabbah S., Erlick C., Shashar N. (2011). Navigation by light polarization in clear and turbid waters. Philos. Trans. R. Soc. B Biol. Sci..

[262] Horváth G., Varjú D. (1995). Underwater refraction-polarization patterns of skylight perceived by aquatic animals through Snell’s window of the flat water surface. Vis. Res..

[263] Cronin T.W., Marshall J. (2011). Patterns and properties of polarized light in air and water. Philos. Trans. R. Soc. B Biol. Sci..

[264] Zhou G., Wang J., Xu W., Zhang K., Ma Z. (2017). Polarization patterns of transmitted celestial light under wavy water surfaces. Remote Sens..

[265] Bai X., Liang Z., Zhu Z., Schwing A., Forsyth D., Gruev V. (2023). Polarization-based underwater geolocalization with deep learning. eLight.

[266] Cheng H., Chen Q., Zeng X., Yuan H., Zhang L. (2023). The polarized light field enables underwater unmanned vehicle bionic autonomous navigation and automatic control. J. Mar. Sci. Eng..

[267] Zhang T., Yang J., Zhao Q., Liu X., Hu P., Yu X., Guo L. (2024). Bio-Inspired Antagonistic Differential Polarization Algorithm for Heading Determination in Underwater Low-Light Environments. IEEE Trans. Ind. Inform..

[268] Moutenet A., Serres J.R., Viollet S. (2023). Ultraviolet vs. Visible Skylight Polarization Measurements. Proceedings of the 2023 IEEE SENSORS.

[269] Labhart T. (1986). The electrophysiology of photoreceptors in different eye regions of the desert ant, Cataglyphis bicolor. J. Comp. Physiol. A.

[270] Liang H., Bai H., Hu K., Lv X. (2023). Bioinspired Polarized Skylight Orientation Determination Artificial Neural Network. J. Bionic Eng..

[271] Poughon L., Aubry V., Monnoyer J., Viollet S., Serres J.R. (2024). A 2 Month-Long Annotated Skylight Polarization Images Database. Recherche Data Gouv, V1. https://entrepot.recherche.data.gouv.fr/dataset.xhtml?persistentId=doi:10.57745/9L2YUB.

[272] Freas C.A., Narendra A., Murray T., Cheng K. (2023). Moonlight Polarisation Pattern Guides Nocturnal Bull Ants Home. bioRxiv.

[273] Zhang Y., Guo L., Yu W., Chen T., Fang S. (2023). Heading determination of bionic polarization sensor based on night composite light field. IEEE Sens. J..

[274] Chen T., Zhang X., Chi X., Hu P., Yu X., Wu H.N., Guo L. (2023). An Autonomous Positioning Method Utilizing Feature Extraction from Polarized Moonlight. IEEE Sens. J..

[275] Wehner R. (2001). Polarization vision—A uniform sensory capacity?. J. Exp. Biol..

[276] Schwind R. (1995). Spectral regions in which aquatic insects see reflected polarized light. J. Comp. Physiol. A.

[277] Wolff L.B., Boult T.E. Polarization/radiometric based material classification. Proceedings of the 1989 IEEE Computer Society Conference on Computer Vision and Pattern Recognition.

[278] Miyazaki D., Kagesawa M., Ikeuchi K. (2004). Transparent Surface Modeling from a Pair of Polarization Images. IEEE Trans. Pattern Anal. Mach. Intell..

[279] Morel O., Stolz C., Meriaudeau F., Gorria P. (2006). Active lighting applied to 3D reconstruction of specular metallic surfaces by polarization imaging. Appl. Opt..

[280] Wolff L.B. (1990). Polarization-based material classification from specular reflection. IEEE Trans. Pattern Anal. Mach. Intell..

[281] Schechner Y., Shamir J., Kiryati N. Polarization-based Decorrelation of Transparent Layers: The Inclination Angle of an Invisible Surface. Proceedings of the IEEE International Conference on Computer Vision (ICCV).

[282] Kalra A., Taamazyan V., Rao S.K., Venkataraman K., Raskar R., Kadambi A. Deep Polarization Cues for Transparent Object Segmentation. Proceedings of the 2020 IEEE/CVF Conference on Computer Vision and Pattern Recognition.

[283] Yu R., Ren W., Zhao M., Wang J., Wu D., Xie Y. (2024). Transparent objects segmentation based on polarization imaging and deep learning. Opt. Commun..

[284] Schwind R. (1984). Evidence for true polarization vision based on a two-channel analyzer system in the eye of the water bug, Notonecta glauca. J. Comp. Physiol. A.

[285] Rankin A.L., Matthies L.H. (2010). Passive sensor evaluation for unmanned ground vehicle mud detection. J. Field Robotics.

[286] Rankin A.L., Matthies L.H., Bellutta P. Daytime water detection based on sky reflections. Proceedings of the 2011 IEEE International Conference on Robotics and Automation.

[287] Nguyen C.V., Milford M., Mahony R. 3D tracking of water hazards with polarized stereo cameras. Proceedings of the 2017 IEEE International Conference on Robotics and Automation.

[288] Berger K., Voorhies R., Matthies L.H. Depth from stereo polarization in specular scenes for urban robotics. Proceedings of the 2017 IEEE International Conference on Robotics and Automation.

[289] Blanchon M., Sidibé D., Morel O., Seulin R., Braun D., Meriaudeau F. P2D: A self-supervised method for depth estimation from polarimetry. Proceedings of the 25th International Conference on Pattern Recognition (ICPR).

[290] Mei H., Dong B., Dong W., Yang J., Baek S.H., Heide F., Peers P., Wei X., Yang X. Glass segmentation using intensity and spectral polarization cues. Proceedings of the IEEE/CVF Conference on Computer Vision and Pattern Recognition.

[291] Olsen R.C., Eyler M., Puetz A., Esterline C. (2009). Initial results and field applications of a polarization imaging camera. Polarization Science and Remote Sensing IV.

[292] Piccardi A., Colace L. (2019). Optical Detection of Dangerous Road Conditions. Sensors.

[293] Li N., Zhao Y., Wu R., Pan Q. (2021). Polarization-guided road detection network for LWIR division-of-focal-plane camera. Opt. Lett..

[294] Xiang K., Yang K., Wang K. (2021). Polarization-driven semantic segmentation via efficient attention-bridged fusion. Opt. Express.

[295] El-Saba A., Bezuayehu T. (2008). Higher probability of detection of subsurface land mines with a single sensor using multiple polarized and unpolarized image fusion. Polarization: Measurement, Analysis, and Remote Sensing VIII; SPIE Defense and Security Symposium.

[296] Zhao Y., Zhang L., Zhang D., Pan Q. (2009). Object separation by polarimetric and spectral imagery fusion. Comput. Vis. Image Underst..

[297] Belskaya I., Cellino A., Gil-Hutton R., Muinonen K., Shkuratov Y., Michel P., Demeo F.E., Bottke W.F. (2015). Asteroid Polarimetry. Asteroids iv.

[298] Ito T., Ishiguro M., Arai T., Imai M., Sekiguchi T., Bach Y.P., Kwon Y.G., Kobayashi M., Ishimaru R., Naito H. (2018). Extremely strong polarization of an active asteroid (3200) Phaethon. Nat. Commun..

[299] Beamer D., Abeywickrema U., Banerjee P. (2018). Statistical analysis of polarization vectors for target identification. Opt. Eng..

[300] Miller M., Blumer R., Howe J. (2002). Active and passive SWIR imaging polarimetry. Polarization Analysis and Measurement IV; International Symposium on Optical Science and Technology, San Diego, CA, USA, 29 July–3 August 2001.

[301] Liang Y., Wakaki R., Nobuhara S., Nishino K. Multimodal material segmentation. Proceedings of the IEEE/CVF Conference on Computer Vision and Pattern Recognition (CVPR).

[302] Lei C., Qi C., Xie J., Fan N., Koltun V., Chen Q. Shape from polarization for complex scenes in the wild. Proceedings of the IEEE/CVF Conference on Computer Vision and Pattern Recognition (CVPR).

[303] Wolff L.B., Boult T.E. (1991). Constraining Object Features Using a Polarization Reflectance Model. IEEE Trans. Pattern Anal. Mach. Intell..

[304] Rahmann S., Canterakis N. Reconstruction of Specular Surfaces Using Polarization Imaging. Proceedings of the IEEE Computer Society Conference on Computer Vision and Pattern Recognition (CVPR).

[305] Miyazaki D., Kagesawa M., Ikeuchi K. Polarization-based transparent surface modeling from two views. Proceedings of the Ninth IEEE International Conference on Computer Vision.

[306] Atkinson G.A., Hancock E.R. (2006). Recovery of surface orientation from diffuse polarization. IEEE Trans. Image Process..

[307] Tozza S., Smith W.A., Zhu D., Ramamoorthi R., Hancock E.R. Linear differential constraints for photo-polarimetric height estimation. Proceedings of the IEEE International Conference on Computer Vision.

[308] Miyazaki D., Saito M., Sato Y., Ikeuchi K. (2002). Determining surface orientations of transparent objects based on polarization degrees in visible and infrared wavelengths. J. Opt. Soc. Am. A.

[309] Huynh C.P., Robles-Kelly A., Hancock E. Shape and refractive index recovery from single-view polarisation images. Proceedings of the 2010 IEEE Computer Society Conference on Computer Vision and Pattern Recognition.

[310] Huynh C.P., Robles-Kelly A., Hancock E.R. (2013). Shape and refractive index from single-view spectro-polarimetric images. Int. J. Comput. Vis..

[311] Mahmoud A.H., El-Melegy M.T., Farag A.A. Direct method for shape recovery from polarization and shading. Proceedings of the 2012 19th IEEE International Conference on Image Processing.

[312] Ngo Thanh T., Nagahara H., Taniguchi R.i. Shape and light directions from shading and polarization. Proceedings of the IEEE Conference on Computer Vision and Pattern Recognition (CVPR).

[313] Smith W.A.P., Ramamoorthi R., Tozza S. (2016). Linear depth estimation from an uncalibrated, monocular polarisation image. Proceedings of the Computer Vision–ECCV 2016: 14th European Conference.

[314] Baek S.H., Jeon D.S., Tong X., Kim M.H. (2018). Simultaneous acquisition of polarimetric SVBRDF and normals. ACM Trans. Graph..

[315] Deschaintre V., Lin Y., Ghosh A. Deep polarization imaging for 3D shape and SVBRDF acquisition. Proceedings of the IEEE/CVF Conference on Computer Vision and Pattern Recognition (CVPR).

[316] Smith W.A., Ramamoorthi R., Tozza S. (2018). Height-from-polarisation with unknown lighting or albedo. IEEE Trans. Pattern Anal. Mach. Intell..

[317] Ba Y., Gilbert A., Wang F., Yang J., Chen R., Wang Y., Yan L., Shi B., Kadambi A., Vedaldi A., Bischof H., Brox T., Frahm J.M. (2020). Deep Shape from Polarization. Proceedings of the Computer Vision—ECCV.

[318] Yang X., Cheng C., Duan J., Hao Y.F., Zhu Y., Zhang H. (2023). Polarized Object Surface Reconstruction Algorithm Based on RU-GAN Network. Sensors.

[319] Ichikawa T., Purri M., Kawahara R., Nobuhara S., Dana K., Nishino K. Shape from sky: Polarimetric normal recovery under the sky. Proceedings of the IEEE/CVF Conference on Computer Vision and Pattern Recognition (CVPR).

[320] Cai Y., Li X., Liu F., Liu J., Liu K., Liu Z., Shao X. (2023). Enhancing polarization 3D facial imaging: Overcoming azimuth ambiguity without extra depth devices. Opt. Express.

[321] Kadambi A., Taamazyan V., Shi B., Raskar R. Polarized 3d: High-quality depth sensing with polarization cues. Proceedings of the IEEE International Conference on Computer Vision (ICCV).

[322] Fukao Y., Kawahara R., Nobuhara S., Nishino K. Polarimetric normal stereo. Proceedings of the IEEE Conference on Computer Vision and Pattern Recognition (CVPR).

[323] Zhu D., Smith W.A.P. Depth From a Polarisation + RGB Stereo Pair. Proceedings of the 2019 IEEE/CVF Conference on Computer Vision and Pattern Recognition (CVPR).

[324] Tian X., Liu R., Wang Z., Ma J. (2022). High quality 3D reconstruction based on fusion of polarization imaging and binocular stereo vision. Inf. Fusion.

[325] Cui Z., Gu J., Shi B., Tan P., Kautz J. Polarimetric multi-view stereo. Proceedings of the IEEE conference on Computer Vision and Pattern Recognition (CVPR).

[326] Cui Z., Larsson V., Pollefeys M. Polarimetric relative pose estimation. Proceedings of the IEEE/CVF International Conference on Computer Vision (CVPR).

[327] Gao D., Li Y., Ruhkamp P., Skobleva I., Wysocki M., Jung H., Wang P., Guridi A., Busam B., Avidan S., Brostow G., Cissé M., Farinella G.M., Hassner T. (2022). Polarimetric Pose Prediction. Proceedings of the Computer Vision—ECCV.

[328] Wang G., Manhardt F., Tombari F., Ji X. Gdr-net: Geometry-guided direct regression network for monocular 6D object pose estimation. Proceedings of the IEEE/CVF Conference on Computer Vision and Pattern Recognition.

[329] Zou S., Zuo X., Wang S., Qian Y., Guo C., Cheng L. (2022). Human Pose and Shape Estimation from Single Polarization Images. IEEE Trans. Multimed..

[330] Huang T., Li H., He K., Sui C., Li B., Liu Y.H. Learning Accurate 3D Shape Based on Stereo Polarimetric Imaging. Proceedings of the IEEE Conference on Computer Vision and Pattern Recognition (CVPR).

[331] Muglikar M., Bauersfeld L., Moeys D.P., Scaramuzza D. Event-based shape from polarization. Proceedings of the IEEE Conference on Computer Vision and Pattern Recognition (CVPR).

[332] Mei H., Wang Z., Yang X., Wei X., Delbruck T. Deep Polarization Reconstruction With PDAVIS Events. Proceedings of the IEEE/CVF Conference on Computer Vision and Pattern Recognition (CVPR).

[333] Jeon D.S., Meuleman A., Baek S.H., Kim M.H. Polarimetric iToF: Measuring High-Fidelity Depth through Scattering Media. Proceedings of the IEEE Conference on Computer Vision and Pattern Recognition (CVPR).

